# Identification of novel inhibitors of dengue viral NS5 RNA-dependent RNA polymerase through molecular docking, biological activity evaluation and molecular dynamics simulations

**DOI:** 10.1080/14756366.2025.2463006

**Published:** 2025-02-12

**Authors:** Keli Zong, Chaochun Wei, Wei Li, Cong Wang, Jiajun Ruan, Xiaojing Liu, Susu Zhang, Hong Yan, Ruiyuan Cao, Xingzhou Li

**Affiliations:** ^a^ College of Chemistry and Life Science, Beijing University of Technology, Beijing, P.R. China; ^b^ Beijing Institute of Pharmacology and Toxicology, Beijing, P.R. China

**Keywords:** DENV-NS5 RdRp, virtual screening, cell-based assays, molecular dynamics simulation, DFT

## Abstract

The DENV-NS5 RNA-dependent RNA polymerase (RdRp) is essential for viral replication, and one of the targets of anti-virus. In this study, the Uni-VSW module was used to virtual screen 1.6 million compounds in the ChemDiv and TargetMol (USA) database, 27 candidates were obtained. Thereby 23 candidates were selected based on their binding free energies by 50 ns MD simulations. The biological activity of the candidates and the reference compounds (**BCX4430** and **Compound 27**) were evaluated on their IC_50_ values against DENV-NGC, CC_50_ values, and selectivity index. Among these, the IC_50_ values of **D1** and **D8** were 13.06 ± 1.17 μM and 14.79 ± 7.76 μM, respectively, which were better than that of **Compound 27** (IC_50_ =19.67 ± 1.12 μM). The comprehensive MD simulations were performed on the candidates to assess the stability behaviour and binding mechanisms. The density functional theory (DFT) analysis was also conducted to explore the structural and electronic properties.

## Graphical Abstract

**Figure:**
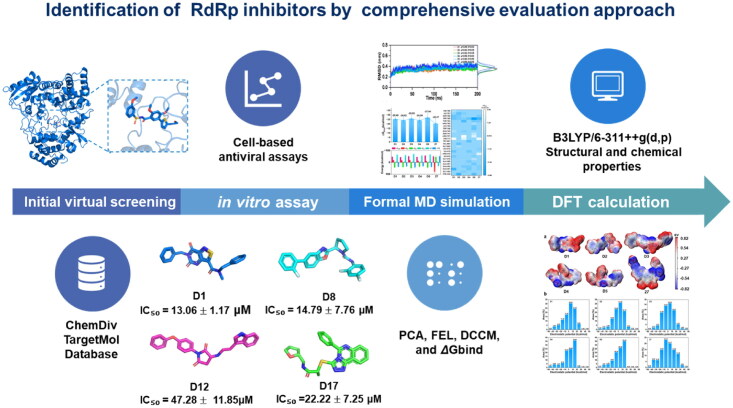

## Introduction

Dengue is a viral disease caused by the dengue virus (DENV), and its prevalence is increasing due to the increased global travel, urbanisation, and inadequate vector control^1–3^. While many infections are asymptomatic or result in mild symptoms, DENV can also lead to severe and fatal conditions, including dengue haemorrhagic fever and dengue shock syndrome^4^^,^^5^. However, there is no specific antiviral therapy, and the treatment relies on supportive care^6^. Compared to biological agents, small molecule therapeutics are particularly beneficial for controlling the outbreaks in low-resource settings due to their ease of production, distribution, and administration^7^. Therefore, there is an urgent need to develop effective small molecule drugs for dengue treatment in response to this widespread and potentially life-threatening viral infection.

**Figure 1.**
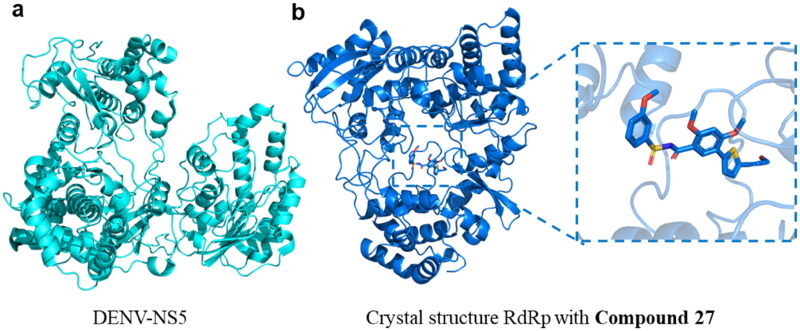
Crystal structure of DENV-NS5 (PDB ID: 4V0R) and crystal structure of DENV-NS5 RdRp domain in complex with **Compound 27** (PDB ID: 5K5M).

The DENV is a positive-stranded RNA virus in the Flaviviridae family consisting of an 11 kb genome^8^. DENV genome encodes three structural proteins, capsid protein C, membrane protein M, and envelope protein E, as well as seven non-structural proteins, including NS1, NS2a, NS2b, NS3, NS4a, NS4b, and NS5^9^^,^^10^. Among these, DENV-NS5 is the most conserved protein within the virus. Its crystal structures, particularly those of the polymerase domain, have been successfully resolved (Figure 1(a))^11^. It has distinct functional domains, in which the C-terminal RNA-dependent RNA polymerase (RdRp) domain facilitates polymerisation (Figure 1(b)) and the N-terminal Methyltransferase (MTase) domain participates in capping. The RdRp domain of DENV-NS5 plays a critical role in viral RNA synthesis, and can minimise concerns about off-target toxicity in host cells for no equivalen in mammaliant^12^. Despite its potential as a drug target, only a limited number of DENV-NS5 RdRp inhibitors have been identified to date, and most do not meet the requirements for development as clinical candidates. This highlights an urgent need to discover novel chemical scaffolds for DENV-NS5 RdRp inhibitors.

**Figure 2.**
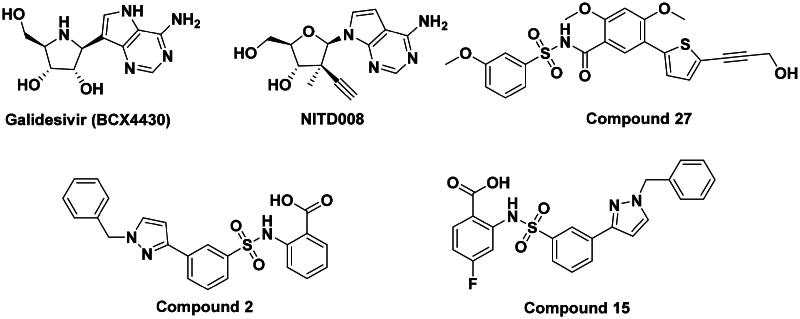
Structures of compounds that inhibit the RdRp domains of DENV-NS5.

The DENV-NS5 RdRp inhibitors were mainly classified into two classes; nucleoside inhibitors (NIs) and non-nucleoside inhibitors (NNIs) shown in Figure 2. To date, there are no inhibitors that have been tested for safety and efficacy in animals and then entered clinical trials. **Galidesivir** (**BCX4430**) was a broad-spectrum antiviral drug that has shown potential to treat various RNA viruses by inhibiting viral RNA polymerase^13^. **NITD008** selectively inhibits flaviviruses replication such as DENV, HCV, YFV, and Powassan virus^14^^,^^15^. **Compound 27** was identified to inhibiting dengue viral RdRp from a fragment hit using a structure-based drug design approach. It was found to bind to a conserved pocket which presented a promising allosteric site for drug discovery in the palm subdomain of RdRp^11^^,^^16^^,^^17^. The 2-[3–(3-methyl-5-oxo-4,5-dihydropyrazol-1yl)benzenesulfonylamino]benzoic acid was identified by high throughput screening (HTS), its derivatives of N-sulfonyl anthranilic acid, such as **Compound 2** and **Compound 15**, were found to interfere with the RdRp domain of DENV-NS5^18^.

## Materials and methods

**Figure 3.**
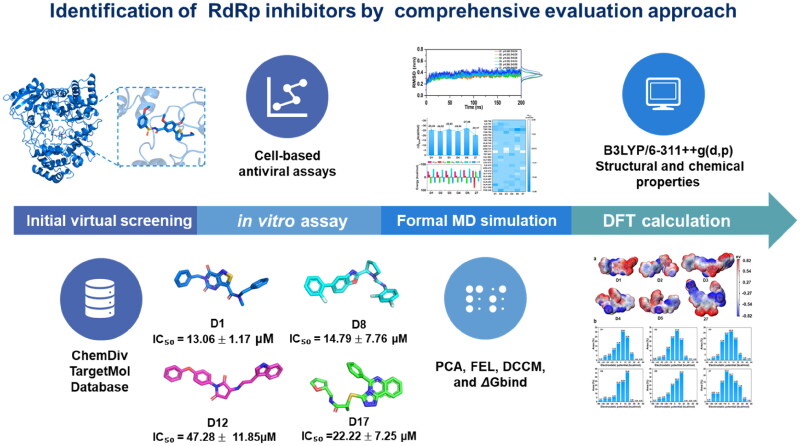
Workflow of this study.

Here, for the allosteric site occupied by **Compound 27**, DENV-NS5 RdRp inhibitors were identified by using molecular docking, molecular dynamics (MD) simulation, cell-based assays, and density functional theory (DFT) calculations. The 1.6 million compounds in ChemDiv and TargetMol (USA) database were screened by a structure-based virtual screening approach. The compounds with favourable interactions to the RdRp allosteric site and good ADMET properties were selected as candidates to inhibiting DENV NS5. According to the 50 ns MD simulation of binding free energy of the docking complex of candidates and the DENV-NS5 RdRp, the selected compounds was evaluated against DENV-NS5 cell-based assays. The 200 ns MD simulation and DFT calculations (B3LYP/6–311++g(d,p)) were carried out on the candidates with higher or similar activity to that of reference **Compound 27**. The results will provide deeper insights into the interaction mechanisms and chemical properties, and be a foundation to identify novel DENV-NS5 RdRp inhibitors with therapeutic potential against the dengue virus. The multi-step workflow was illustrated in Figure 3.

### Protein preparation

The DENV-NS5 RdRp protein (PDB ID: 5K5M) was prepared and focused on optimising the structure for molecular docking and virtual screening. The A chain (residues 273–889) was retained, with water molecules within 6.00 Å of original ligand preserved to maintain potential stabilising interactions by protein preparation module (Hermite platform: https://hermite.dp.tech, DP Technology). Critical components such as zinc ions and other heteroatoms were also retained to preserve the protein’s native configuration. Then, missing residues and side chains were rebuilt, and disulphide bonds were generated to enhance structural stability. The protonation state was adjusted to pH 7.4 ± 2.0, ensuring accurate modelling of ionisable residues, followed by hydrogen bond network optimisation to promote favourable binding interactions. Finally, energy minimisation was performed to resolve steric clashes and ensure a relaxed, biologically relevant structure, preparing the protein for precise interaction studies.

### Ligand preparation

The ligands were compounds from the ChemDiv (https://www.chemdiv.com/) and TargetMol (USA) (https://www.targetmol.cn/) database to ensure comprehensive exploration of chemical diversity. To account for stereochemical variation, both chiral and cis-/trans- isomers were generated, with key chiral centres retained while allowing variation at other stereo enters, yielding up to 8 isomers by ligand preparation module (Hermite platform: https://hermite.dp.tech, DP Technology). Protonation states were adjusted to reflect physiological pH (7.4), ensuring biologically relevant ionisation forms. Additionally, 8 conformations per isomer per ionisation state were generated to explore a wide range of potential binding poses. The workflow also incorporated ring conformation sampling and polypeptide optimisation to account for molecular flexibility. This rigorous preparation ensures the ligands are structurally diverse, properly ionised, and conformationally optimised, thereby enhancing the reliability of subsequent virtual screening and docking analyses in identifying high-affinity candidates.

### Virtual screening

A multi-tiered virtual screening strategy was employed to identify high-affinity ligands, with each stage designed to refine and optimise the candidate pool by Uni-VSW module (Hermite platform: https://hermite.dp.tech, DP Technology). The initial screening began with fast mode docking, advancing the top 5% of ligands for further evaluation. Subsequently, balanced mode docking was performed on these shortlisted compounds, retaining the top 5% for detailed analysis. Finally, detailed mode docking was carried out using the same criteria to ensure precision in identifying the most promising candidates. The vina scoring function was applied consistently across all docking stages to evaluate binding affinities. To further optimise ligand poses, Uni-Mol was used for conformational refinement, and rescoring with Uni-Score ensured accurate ranking of ligand-protein interactions. Binding free energy calculations were performed using Molecular Mechanics/Generalized Born Surface Area (MM/GBSA) with the Amber 03 force field for the protein and general amber force field 2 (GAFF2) force field for the ligands. The solvation mode was set to GBSA with a dielectric constant of 4.00 and implicit solvent dielectric of 80.00. To account for conformational changes upon binding, energy minimisation was applied during the MM/GBSA calculations, ensuring stability of the complexes and reliability of the binding affinity predictions. This systematic approach enabled the efficient identification of potential high-affinity ligands for further experimental validation.

### ADMET prediction

The pharmacokinetic properties of the candidate compounds were predicted using the pkCSM tool (https://biosig.lab.uq.edu.au/pkcsm/prediction), which enables comprehensive absorption, distribution, metabolism, excretion, and toxicity (ADMET) profiling. Molecular structures were provided *via* SMILES strings or batch file uploads containing molecule identifiers. The pkCSM platform employs graph-based signatures and all-pair shortest path algorithms to predict pharmacokinetic behaviour, generating detailed insights into drug-like properties. This tool facilitated the identification of compounds with favourable ADMET profiles, streamlining the selection process for further experimental validation and optimisation in drug development.

### MD simulations

The GROMACS 2020.7 beta version was utilised to conduct all-atom MD simulations to study the binding interactions and stability of protein-compound complexes. The simulations were performed over a duration of 200 ns at 298.15 K, applying the Amber ff14SB force field^19^. Ligand parameters were prepared using the General Amber Force Field (GAFF)^20^, and atomic charges were derived at the B3LYP/cc-pVDZ level with the assistance of Gaussian 16^21^, Multiwfn 3.8^22^, and Sobtop tools^23^. Each simulation system was solvated in a 10 Å cubic box filled with TIP3P water molecules, and counter ions (Na^+^ and Cl^-^) were added using the GROMACS genion module to neutralise the systems. Energy minimisation was completed under periodic boundary conditions with the steepest descent algorithm over 5000 steps, while long-range electrostatic interactions were calculated using the Particle Mesh Ewald (PME) method. System equilibration was conducted in two stages: first, the temperature was gradually increased from 0 K to 298.15 K under the NVT ensemble using the V-rescale thermostat; second, the NPT ensemble with the Parrinello-Rahman barostat was employed to maintain a stable pressure of 1 atm. For analysis, MD simulations lasting 50 ns were used to generate snapshots for Molecular Mechanics/Poisson Boltzmann Surface Area (MM/PBSA) calculations and to identify virtual screening hits. Additionally, longer MD simulations of 200 ns were carried out to investigate the interaction mechanisms between candidate molecules and the DENV-NS5 RdRp protein.

### DFT calculations

The density functional theory (DFT) was used to study structural, physical, and chemical properties of compounds by Gaussian 16 program and visualisation conducted using VMD^24^. The structural coordinates of the compounds, characterised by a singlet state and neutral charge, were optimised using the B3LYP/6–311++G(d,p) level of theory. No symmetry constraints were applied during the optimisation process. The molecular electrostatic potential (MEP)^25^, interaction region indicator (IRI)^26^, and HOMO-LUMO energy levels of compounds were calculated by utilising Multiwfn software.

### Cell-based antiviral assays

In this study, the DENV2 NGC strain (provided by Robert B. Tesh, University of Texas Medical Branch) and BHK-21 cells (Baby hamster kidney cells, ATCC CCL10) were used for cell-based antiviral evaluations. Viral stocks were prepared by infecting C6/36 cells (Aedes albopictus clone C6/36; ATCC CRL166), cultured in RPMI 1640 medium (Invitrogen, Burlington, ON, Canada) containing 2% foetal bovine serum (FBS) (Sigma-Aldrich, Shanghai, China) and 0.5% penicillin-streptomycin. The infected cultures were incubated at 28 °C with 5% CO_2_ for seven days until cytopathic effects (CPE) were detected. Supernatants were collected by centrifuging at 2,000 g for 5 min to remove debris, mixed with 20% FBS, and stored in aliquots at −80 °C. Viral titres were quantified using the Promega Viral ToxGlo assay (Fisher Scientific, Ottawa, ON, Canada) on BHK-21 cells, and the 50% tissue culture infective dose (TCID_50_) was determined following the manufacturer’s instructions. To test antiviral activity, BHK-21 cells were seeded in 96-well plates at 2,500 cells per well using Dulbecco’s modified Eagle’s medium (DMEM) with 10% FBS and 1% penicillin-streptomycin. After overnight incubation at 37 °C, cells were exposed to 100 TCID_50_ of DENV for 90 min. Following infection, the medium was replaced with DMEM supplemented with 2.5% FBS and different concentrations of the test compounds. Cultures were maintained at 37 °C with 5% CO_2_ for 3 days. Fresh Viral Tox reagent was prepared, and 100 μL was added to each well before incubating the plates for at least 10 min. Luminescence readings were taken using a 1450 MicroBeta TriLux scintillation counter (PerkinElmer). Data were analysed by plotting luminescence values against logarithmic compound concentrations, and nonlinear regression was carried out using GraphPad Prism 10.0 software.

## Results and discussion

### Virtual screening

Firstly, the docking protocol has been validated by redocking original ligand (**Compound 27**) of crystal structure of DENV-NS5 to its binding domain. The docked pose is in agreement with the original pose, and the RSMD value of the two is 0.346 Å (Figure S1). After systematic molecular docking process, 200 candidates were retained from the 1.6 million compounds in the ChemDiv and TargetMol (USA) database. Their performance was evaluated using three key metrics: docking score, Uni-Score Rescore, and MM/GBSA binding free energy. Docking Score provided a quick estimation of the binding affinity by calculating the fit of the ligand in the receptor’s binding site. It served as a preliminary filter to identify potentially active compounds. Uni-Score Rescore refined the initial docking results by optimising the ligand’s pose and evaluating more detailed molecular interactions, ensuring accurate ranking of compounds. This rescoring step helped to reduce false positives from the initial docking stage. MM/GBSA binding free energy offered a more precise calculation of the binding affinity by incorporating energy minimisation and solvation effects, accounting for conformational changes that occur upon ligand binding. Using these three metrics together ensured a comprehensive evaluation of each compound, balancing speed, accuracy, and molecular detail.

To narrow down the selection of 200 compounds, a cut-off criterion based on three key parameters: Score (−6 kcal/mol), Uni-score Rescore (−7 kcal/mol), and MM/GBSA binding free energy (−2 kcal/mol), were applied. Using these thresholds, a subset of 27 candidate compounds was identified for 50 ns MD simulations with the reference **Compound 27** (**Table S1)**. Most candidate compounds demonstrated stable RMSD values after the initial equilibration phase, indicating consistent binding interactions throughout the simulation (Figure S2). The binding free energy for each complex was calculated using the MM/PBSA method based on the final 5 ns of the simulation trajectories. Among the 27 candidates, 23 compounds exhibited binding free energy values superior to that of the reference **Compound 27**, highlighting their potential as promising inhibitors (Figure S2 and Table S2).

Evaluating the ADMET properties of candidate compounds was essential for ensuring their potential success as drug candidates. The ADMET properties of candidate compounds were evaluated to identify favourable pharmacological profiles with the reference **BCX4430**, **NITD008**, and **Compound 27** (**Table S3**). Water solubility values (log mol/L) for the candidates varied between −2.18 (**D15**) and −5.39 (**D21**), indicating a wide range of solubility characteristics. **D21** displayed the lowest water solubility, while **BCX4430** showed significantly higher solubility (−2.48) than most candidates, suggesting its advantage in aqueous environments. **Compound 27** exhibited moderate solubility (−4.79), consistent with its role as a reference. In terms of Caco2 permeability, compounds such as **D8** (1.29) and **D20** (1.231) exhibited excellent permeability, outperforming **BCX4430** (0.51) and **Compound 27** (−0.03). Intestinal absorption values were notably high for most candidates, with **D17** achieving the highest absorption (96.73%), whereas **BCX4430** displayed significantly lower absorption (53.42%). **Compound 27** showed an intermediate absorption level of 81.39%. Regarding distribution, candidates such as **D13** (0.56) and **D12** (0.47) showed favourable VDss values, indicating better tissue distribution compared to **BCX4430** (0.72) and **Compound 27** (−0.54). Hepatotoxicity was predicted for many candidates, including **D3**, **D4**, and **D6**, as well as the reference **Compound 27**, while **BCX4430** was not flagged for hepatotoxicity. All compounds exhibited high intestinal absorption and acceptable water solubility. Toxicity evaluation revealed that compounds such as **D1** and **D17** were predicted to be non-hepatotoxic and non-hERG I inhibitors, providing a safety advantage over **Compound 27**, which was identified as both hepatotoxic and a potential hERG I inhibitor. Furthermore, chronic oral toxicity predictions showed that compounds like **D1** (1.22 log mg/kg_bw/day) and **D12** (1.29 log mg/kg_bw/day) had acceptable profiles compared to **Compound 27** (1.15 log mg/kg_bw/day). Overall, the compounds exhibited superior pharmacokinetic profiles and safety compared to the reference compounds.

### Biological activity assessment

The biological activity of the candidates and the reference compounds (**BCX4430** and **Compound 27**) was evaluated based on their IC_50_ values against DENV-NGC, CC_50_ values, and selectivity index (SI**)**, as shown in Table 1. These parameters provided insights into the antiviral potency, cytotoxicity, and selectivity of the compounds. Among the candidate compounds, **D1** exhibited the most potent antiviral activity with an IC_50_ of 13.06 ± 1.17 μM, followed closely by **D8 (**14.79 ± 7.76 μM**)**. Compound **D17** showed moderate activity (22.22 ± 7.25 μM), while **D12** displayed lower potency with an IC_50_ of 47.28 ± 11.85 μM, the reference **Compound 27** with an IC_50_ of 19.67 ± 1.12 μM, the **BCX4430** with an IC_50_ values of 41.59 ± 2.95 μM. The candidate compounds displayed varying levels of cytotoxicity. **D12** exhibited the highest CC_50_ **(**163.3 ± 14.14 μM**)**, indicating low toxicity, followed by **D2 (**99.20 ± 1.93 μM**)**. In contrast, **D1** and **D17** showed lower CC_50_ values of 37.47 ± 2.03 μM and 52.95 ± 10.54 μM, respectively. Among the reference compounds, **Compound 27** had a CC_50_ of 26.87 ± 3.02 μM, while **BCX4430** showed a CC_50_ of 100.24 ± 12.45 μM. The SI was calculated as the ratio of CC_50_ to IC_50_, reflected the therapeutic window of each compound. **D8** showed the highest SI among the candidates, with a value of 6.71, indicating high selectivity for the virus over host cells. **D1**, **D12**, and **D17** exhibited moderate SI values of 2.87, 3.45, and 2.38, respectively, and **BCX4430** displayed a lower SI of 1.29. Overall, the results of biological activity revealed that the **D1** and **D8** exhibited higher antiviral activity than that of the reference **Compound 27**. **D12** and **D17** demonstrated antiviral activity comparable to that of **BCX4430**.

**Table 1.** Bioactivity evaluation results of the active compounds.

Compound	Structure	^a^DENV-NGC-IC_50_ (μM)	^a^CC_50_(μM)	SI (selectivity index)
**D1**	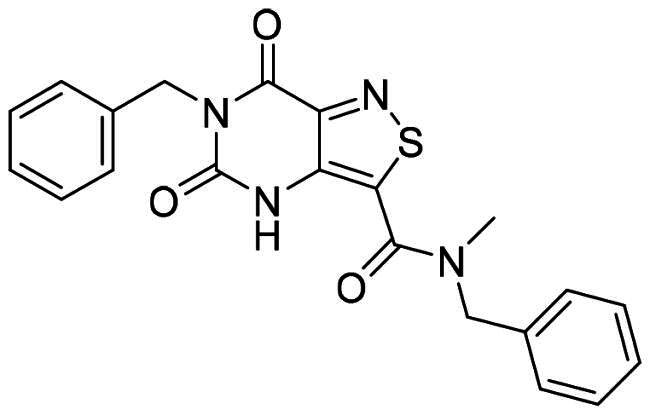	13.06 ± 1.17	37.47 ± 2.03	2.87
**D3**	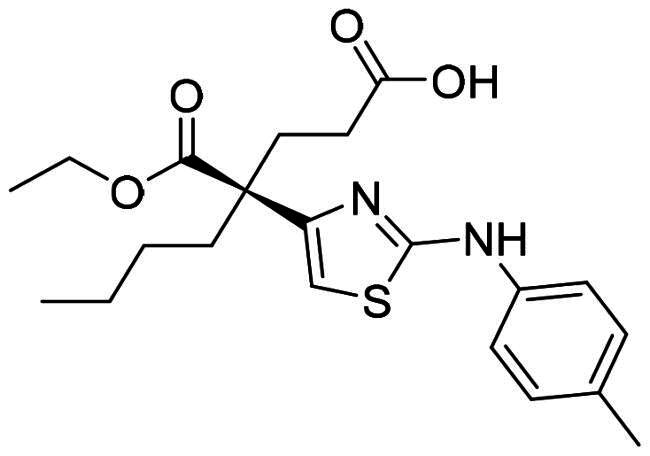	>66.67	–	–
**D4**	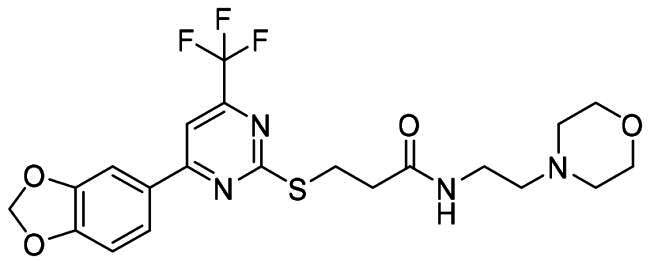	>200	–	–
**D5**	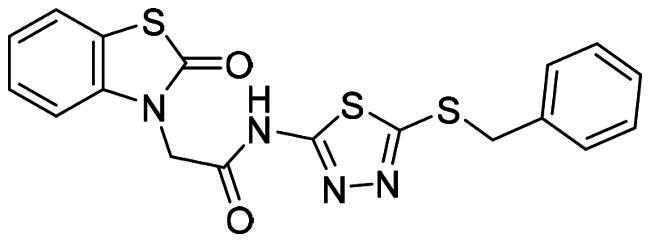	28.53(>66.67)	>200	–
**D6**	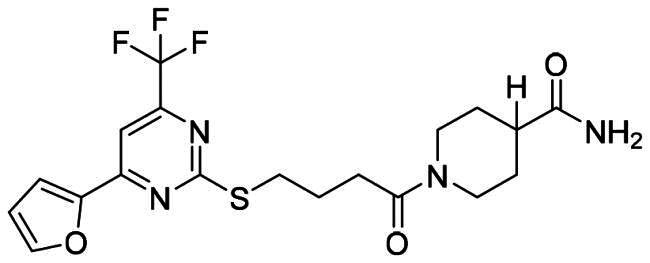	>200	–	–
**D7**	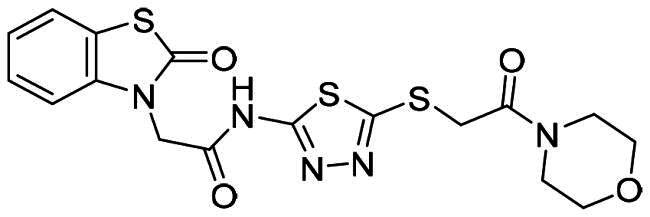	>200	–	–
**D8**	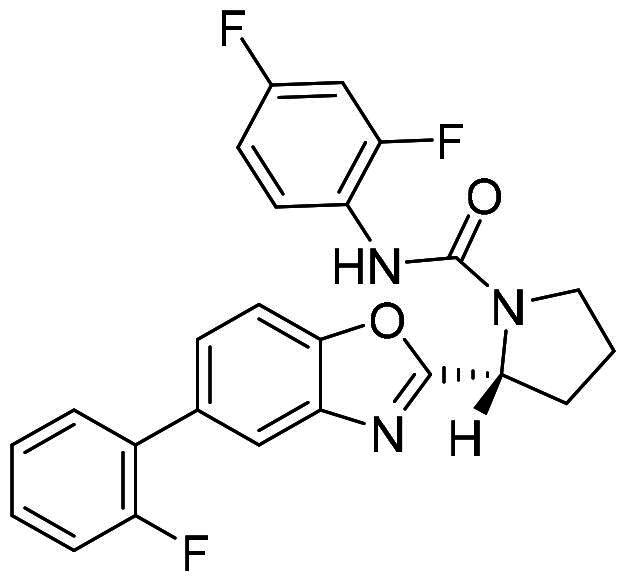	14.79 ± 7.76	99.20 ± 1.93	6.71
**D9**	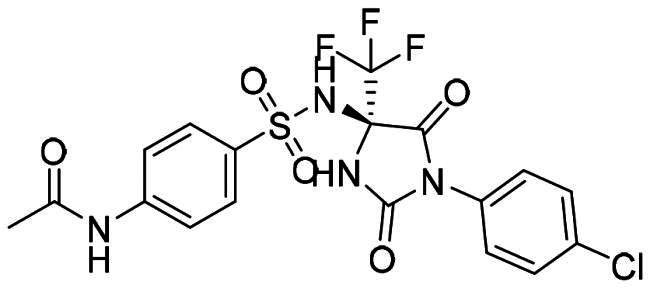	>200	–	–
**D10**	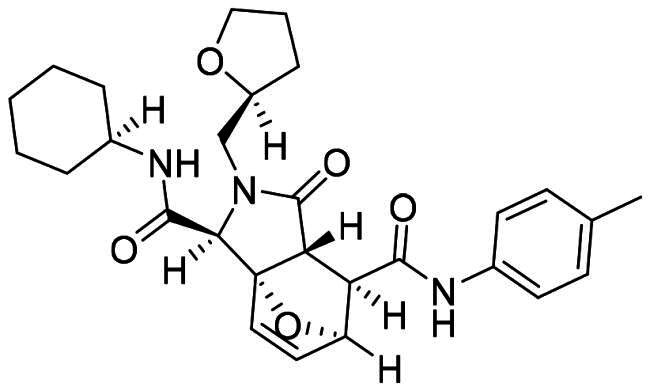	>200	–	–
**D11**	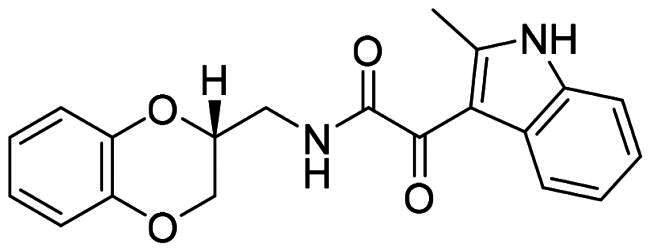	>66.67	87.29 ± 2.75	>1.31
**D12**	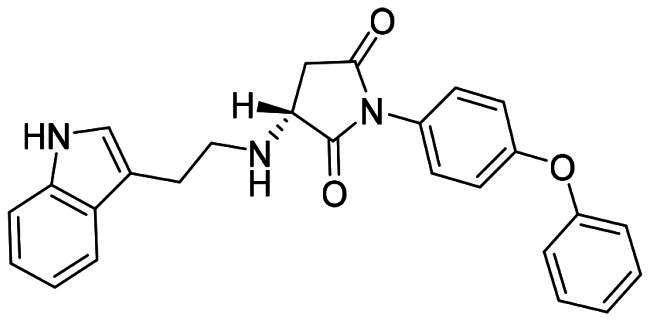	47.28 ± 11.85	163.3 ± 14.14	3.45
**D13**	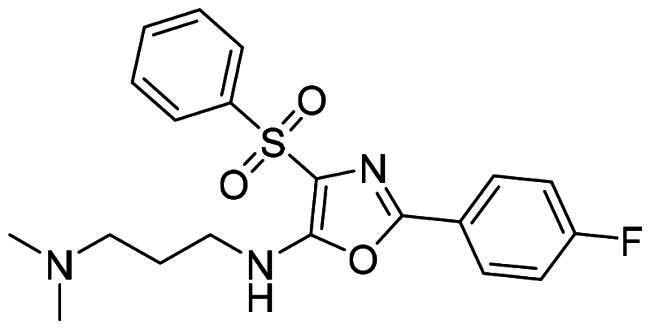	19.91(>200)	–	–
**D14**	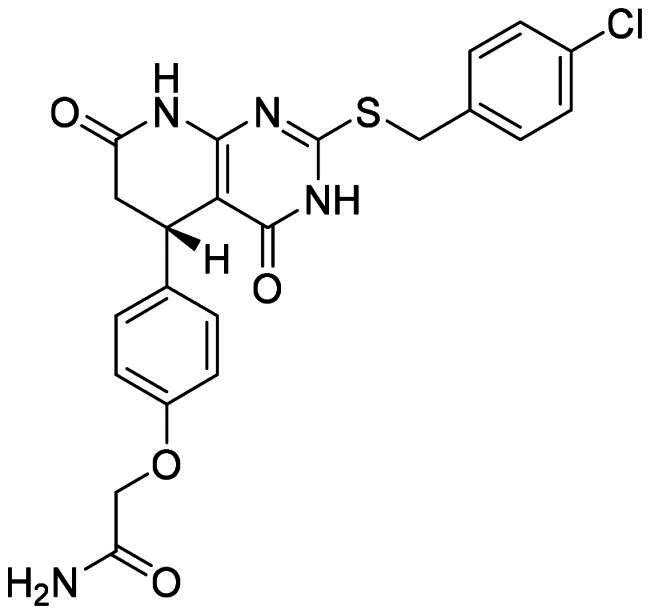	31.24(>200)	>200	–
**D15**	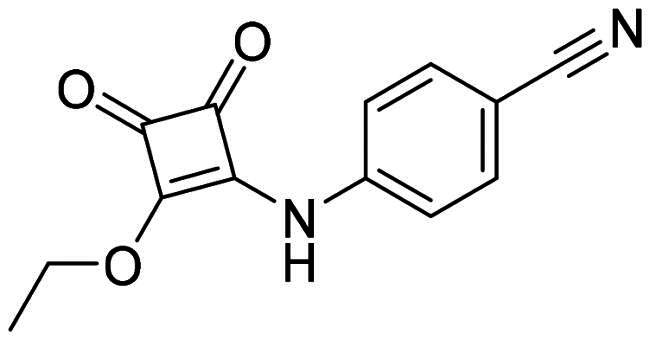	>200	–	–
**D16**	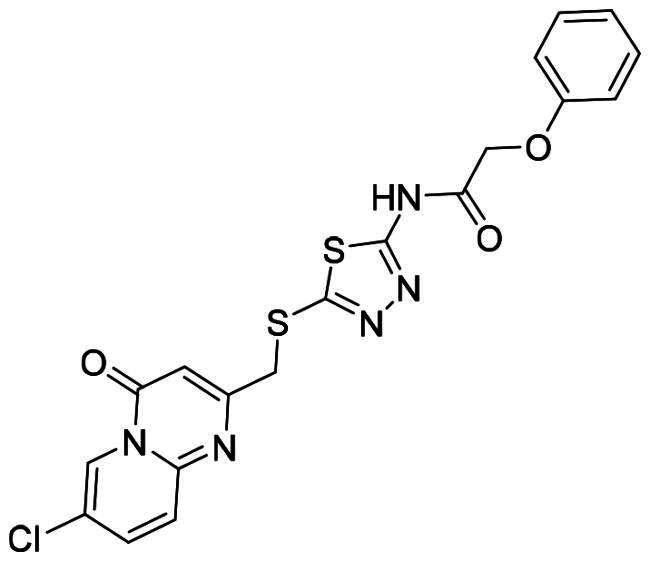	>200	–	–
**D17**	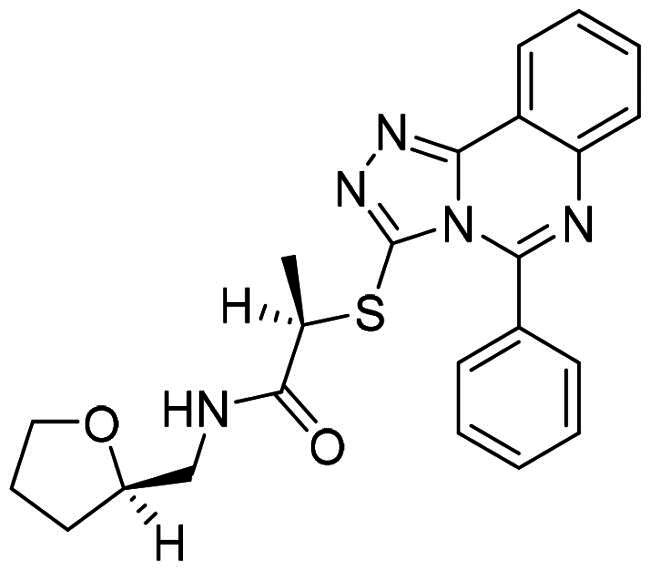	22.22 ± 7.25	52.95 ± 10.54	2.38
**D18**	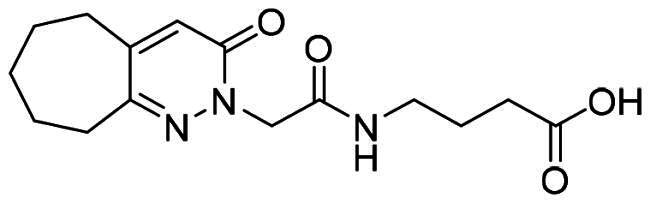	>200	–	–
**D19**	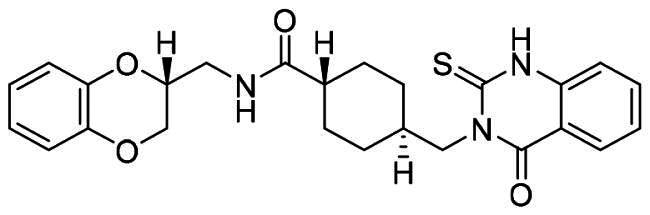	>200	–	–
**D20**	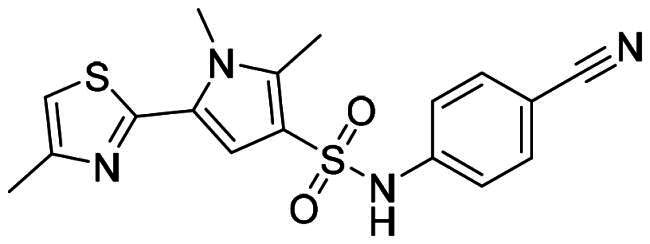	13.92(>200)	44.47(>200)	–
**D21**	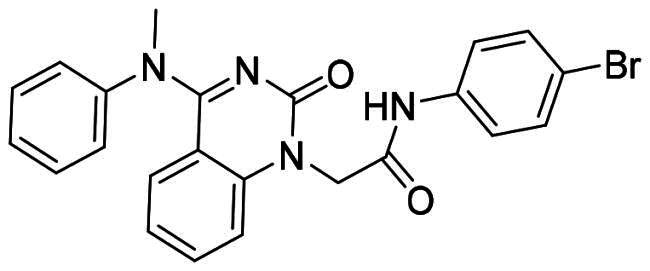	>200	–	–
**D23**	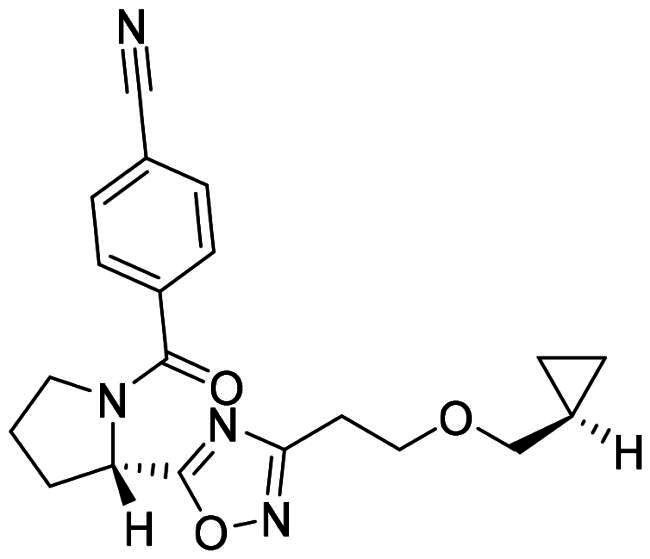	122.79(>200)	>200	–
**D24**	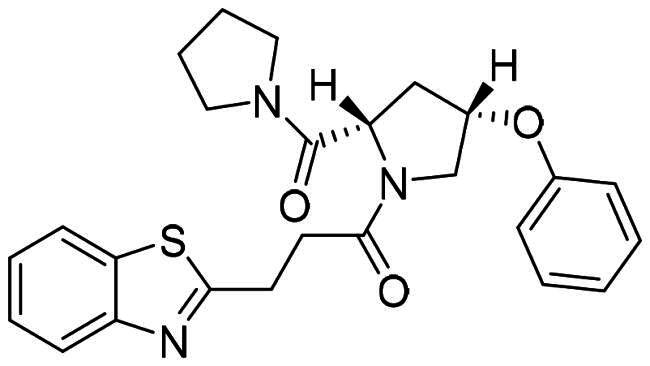	>66.67	>200	>2.30
**D26**	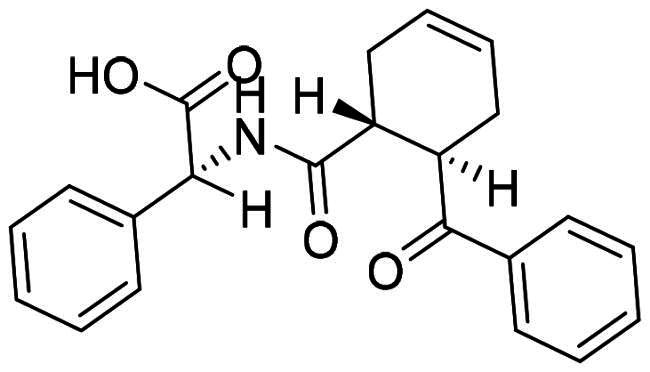	>200	–	–
**BCX4430**	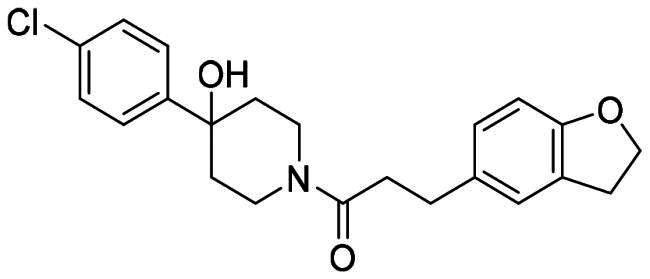	41.59 ± 2.95	100.24 ± 12.45	2.40
**27**	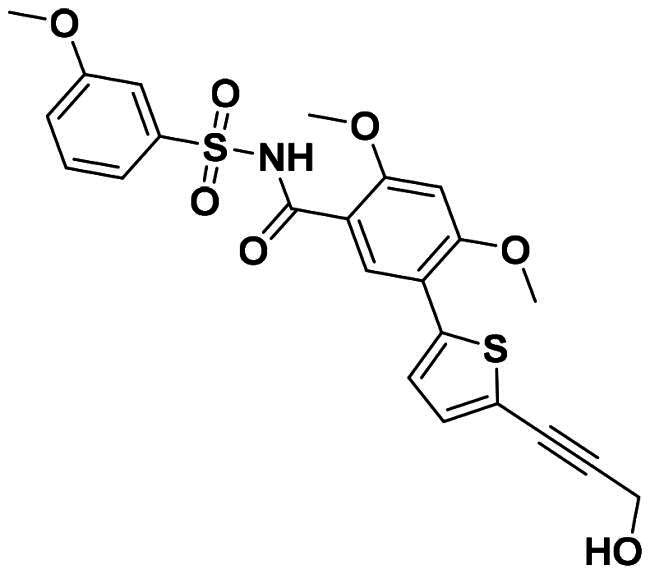	19.67 ± 1.12	26.87 ± 3.02	1.37

^a^IC_50_ values against DENV-NGC represent the mean ± SD of two individual experiments.

### MD Simulation analysis

MD simulations were performed to analyse the structural stability and flexibility of the complexes formed between the target protein and the candidate compounds. Based on the comprehensive computational and pharmacological analysis, the docked complexes of DENV-NS5 RdRp with four candidate compounds (**D1**, **D8**, **D12**, and **D17**) and the reference **Compound 27** were selected for all-atom MD simulations. These simulations aimed to assess the stability and dynamic behaviour of the complexes, providing deeper insights into their potential as effective inhibitors. To ensure the reliability and robustness of the results, three independent MD simulations were performed for each system. The RMSD values obtained from all three trajectories were highly consistent, demonstrating the accuracy and reproducibility of the simulations.

#### Structural stability analysis

**Figure 4.**
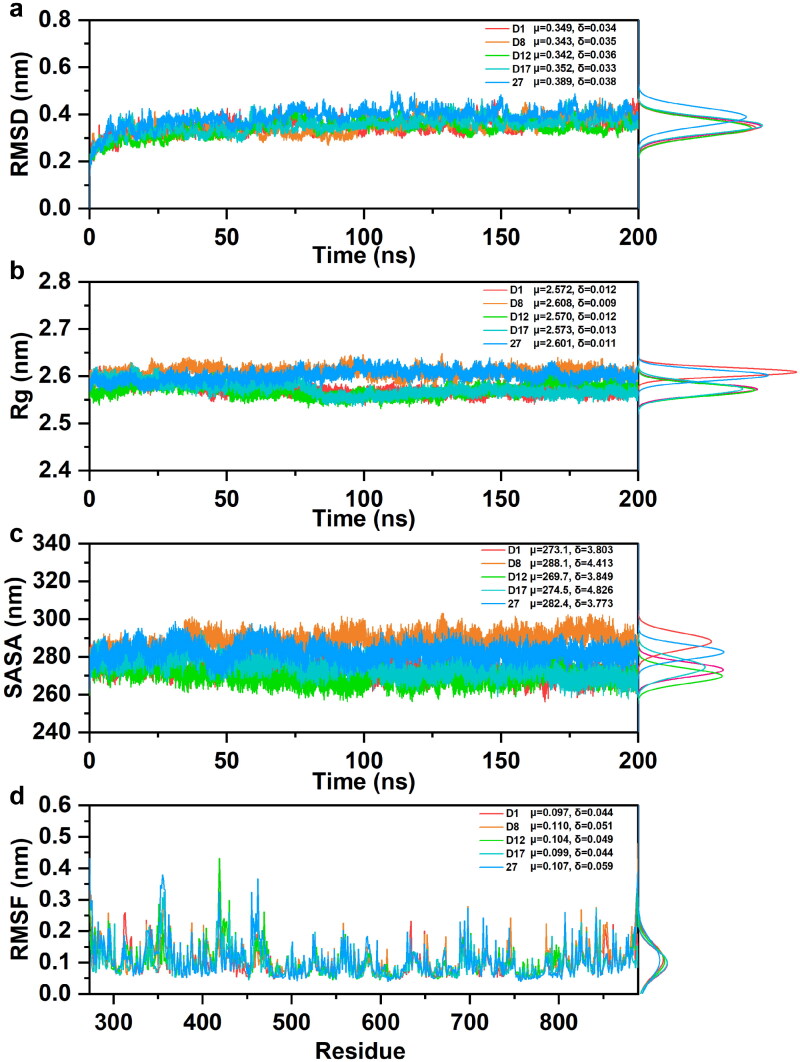
(a) RMSD of candidate compounds and **Compound 27** complexes. (b) SASA of candidate compounds and **Compound 27** complexes. (c) Rg of candidate compounds and **Compound 27** complexes. (d) RMSF of candidate compounds and **Compound 27** complexes over last 50 ns.

The RMSD values, shown in Figure 4(a), indicated the stability of each complex over time. The average RMSD values of the candidate compounds ranged from 0.343 nm (**D8**) to 0.352 nm (**D17**), and **Compound 27** exhibited a slightly higher RMSD value of 0.389 nm, suggesting that the candidate compounds generally formed more stable complexes than **Compound 27**. The low standard deviations in all cases indicated consistent stability throughout the simulation. The Rg values, presented in Figure 4(b), reflected the compactness of the protein-ligand complexes over time. The average Rg values for the candidate compounds ranged between 2.570 nm (**D12**) and 2.582 nm (**D1**), whereas **Compound 27** exhibited an average Rg of 2.639 nm. The higher Rg value of **Compound 27** suggested a less compact complex, while the candidate compounds maintained more compact and stable structures. SASA analysis, illustrated in Figure 4(c), revealed the exposure of the complexes to the solvent environment. The average SASA values for the candidate compounds varied from 269.7 nm^2^ (**D12**) to 288.1 nm^2^ (**D8**), with **Compound 27** showing a slightly lower value of 282.4 nm^2^. The differences in SASA indicated minor variations in the surface exposure, but all complexes remained within a comparable range, suggesting that the candidates maintained stable solvent interactions similar to **Compound 27**.

The RMSF values, depicted in Figure 4(d), provided insights into the flexibility of individual residues in the protein-ligand complexes. The candidate compounds exhibited average RMSF values between 0.097 nm (**D1**) and 0.104 nm (**D12**), while the reference **Compound 27** had a similar average RMSF of 0.107 nm. The comparable RMSF values across all complexes indicated that the flexibility of the protein was not significantly affected by the presence of different ligands. The MD simulation results suggested that the candidate compounds formed stable and compact complexes with the target protein with lower RMSD and Rg values than those of the reference **Compound 27**. The SASA and RMSF analyses showed that the candidates exhibited similar solvent accessibility and flexibility to **Compound 27**. The overall stability of the candidate complexes, as indicated by the RMSD, Rg, SASA, and RMSF metrics, suggested that these compounds are promising alternatives to **Compound 27** for further experimental validation.

#### PCA analysis

**Figure 5.**
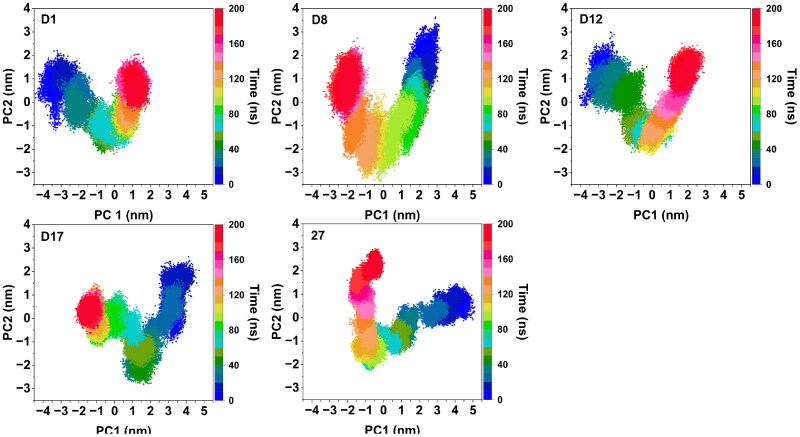
Representation of the 2D projections of candidate compounds and **Compound 27** complexes conformational changes during the simulation.

The 2D projections of the conformational changes of the protein-ligand complexes during the MD simulations were analysed using principal component analysis (PCA). The projections of the first two principal components (PC1 and PC2) for the four candidate compounds and the reference **Compound 27** were shown in Figure 5. These PCA plots revealed the extent of conformational sampling and the dynamic behaviour of the complexes over the simulation time. **D1** and **D12** exhibited relatively constrained conformational spaces, indicating that their structural flexibility was limited during the simulation. This behaviour suggested that these compounds maintained more rigid binding interactions with the protein target, which might have enhanced binding stability. In contrast, **D8** and **D17** displayed conformational dynamics that closely resembled those of **Compound 27**, as evidenced by their broader PCA projections. This similarity suggested that **D8** and **D17** exhibited greater flexibility in their binding interactions, and akin to **Compound 27**. Such flexibility might have enabled these compounds to accommodate the inherent structural changes of the protein, potentially improving their binding adaptability. The PCA analysis highlighted distinct differences in the conformational behaviour of the selected candidates. **D1** and **D12**’s restricted conformational variability indicated stable but rigid binding interactions, whereas **D8** and **D17**’s dynamic profiles, resembling that of **Compound 27**, suggested a balance of stability and flexibility.

#### FEL and DCCM analysis

**Figure 6.**
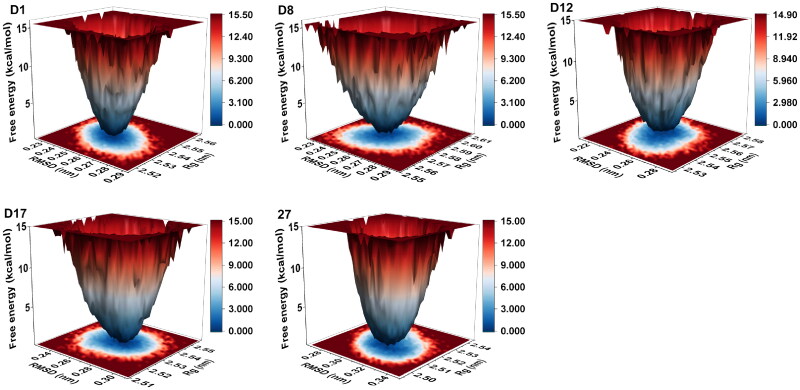
FEL of candidate compounds and **Compound 27**.

The free energy landscapes (FEL) of the candidate compounds and the reference **Compound 27** were analysed to assess the conformational stability and energy distribution of their complexes throughout the MD simulations were shown in Figure 6. Each FEL plot represented the relationship between RMSD, Rg, and free energy (kcal/mol), providing insights into the preferred conformational states and stability of the complexes. The FEL analysis revealed differences in the conformational stability of the candidate compounds compared to the reference **Compound 27**. **D1** displayed the deepest and narrowest energy well, indicating the most stable conformational states with minimal fluctuations. **D8** exhibited a deeper but broader energy well, suggesting a balance between stability and moderate conformational flexibility. In contrast, **D12** and **D17** demonstrated energy landscapes resembling that of **Compound 27**, with shallower energy wells indicating greater conformational flexibility and reduced stability compared to **D1** and **D8**. Overall, the FEL plots revealed that **D1** formed the most stable and compact complex, followed by **D8**, while **D12** and **D17** showed dynamic behaviours similar to **Compound 27**. The superior stability and favourable energy profiles of **D1** and **D8** highlight their potential as promising alternatives to **Compound 27**.

**Figure 7.**
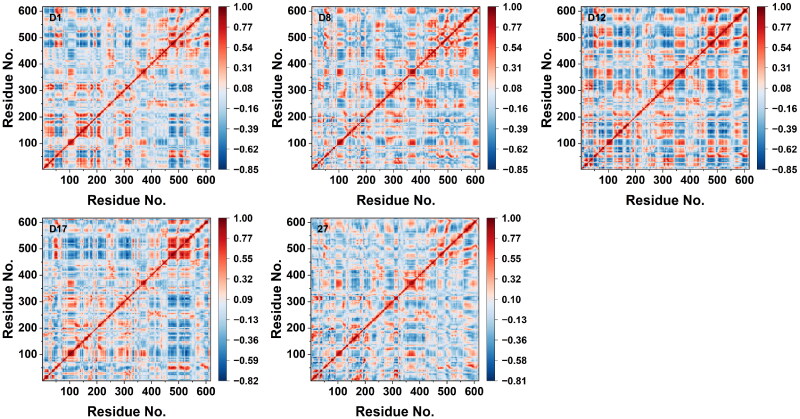
DCCM of candidate compounds and **Compound 27**.

The dynamic cross-correlation matrices (DCCM) of the candidate compounds and the reference **Compound 27** were analysed to evaluate the correlated motions between residues during the MD simulations, and shown in Figure 7. The correlation values ranged from −1 to +1, where positive correlations (red) indicated coordinated motions between residues, and negative correlations (blue) reflected anti-correlated motions. The DCCM plots of **D1**, **D12**, and **D17** exhibited more extensive regions with positive correlations along the diagonal, suggesting stronger coordinated motions within these complexes. This indicated that these candidate compounds stabilised collective residue motions. On the other hand, **D8** showed weaker correlations and more dispersed patterns, suggesting greater structural flexibility and less coordinated motion within these complexes. In comparison, the DCCM of **Compound 27** exhibited fewer and more scattered positive correlations, indicating less stable coordinated motion across the residues. Overall, the candidate compounds, particularly **D1**, **D12**, and **D17**, demonstrated stronger coordinated residue motions with more stable binding interactions compared to the reference **Compound 27**.

### Clustering and protein-ligand interaction analysis

**Figure 8.**
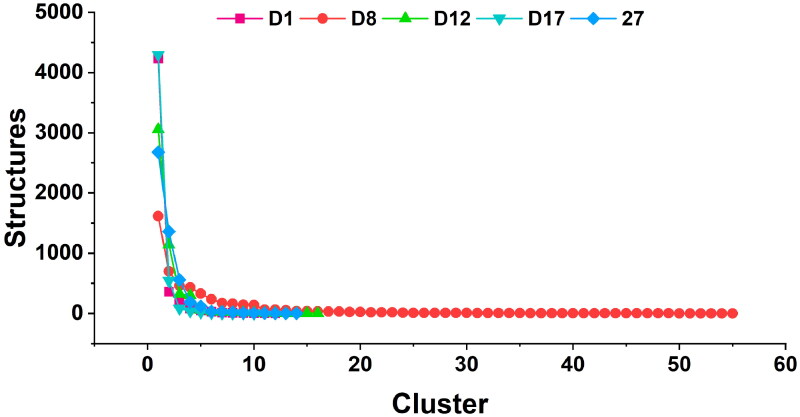
Cluster analysis of the last 50 ns of the simulation.

The cluster analysis of the last 50 ns of the MD simulation, shown in Figure 8, was performed to assess the structural diversity of the candidate compounds compared to the reference **Compound 27**. The number of distinct clusters reflected the conformational variability of each complex, with a higher number of clusters indicating greater structural diversity. The candidate compounds **D1**, **D12**, and **D17** formed fewer clusters, indicating more stable and convergent conformations. In contrast, **D8** exhibited slightly higher structural diversity, reflecting greater flexibility compared **Compound 27**.

**Figure 9.**
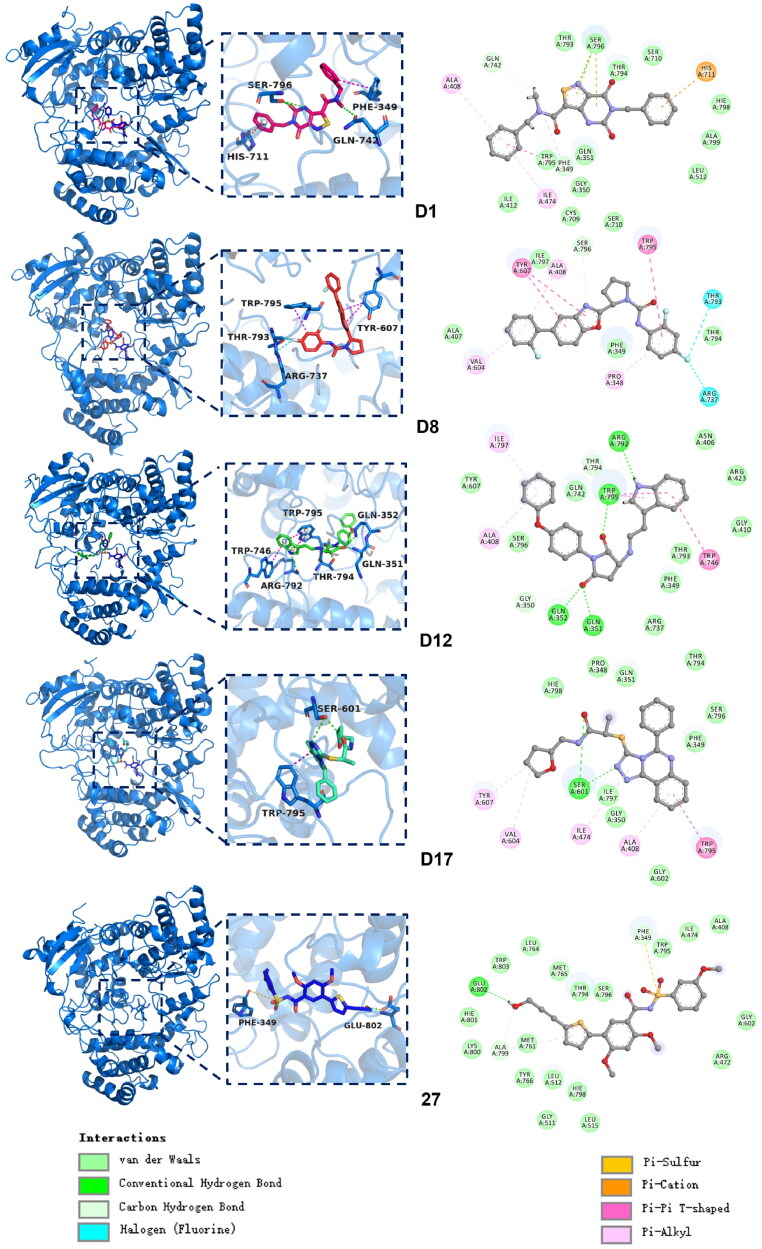
Protein-ligand interactions of candidate compounds and **Compound 27.** (Left: the three-dimensional stacking diagram and enlarged diagram of candidate compounds and **Compound 27**. Right: the two-dimensional diagram of the interaction).

The protein-ligand interactions of the candidate compounds and the reference **Compound 27** were analysed to understand their binding modes within the target binding site. The representative structure was extracted from the largest conformational cluster to analyse its interactions. The three-dimensional stacking and two-dimensional interaction diagrams for each compound were illustrated in Figure 9, showing various key interactions that contributed to binding stability. The candidate compounds exhibited a range of interactions, including hydrogen bonds, van der Waals interactions, π-stacking interactions, and hydrophobic contacts. Notably, **D1** formed multiple hydrogen bonds with residues SER796 and GLN742, and additional π-π interactions with PHE349, contributing to strong binding stability. And **D8** formed halogen bonds with THR793 and ARG737, indicating a unique interaction pattern compared to other candidates. Additionally, **D8** engaged in π-π interactions with TRP795 and TYR607, which could contribute to its stability within the binding site. **D12** demonstrated distinct interactions, engaging with GLN351, GLN352, ARG792, and TRP795, with multiple hydrogen bonds stabilising the complex. It also interacted π-π interactions with TRP746. **D17** formed significant interactions with TRP795 and SER601, involving π-π interactions and hydrogen bonds. These interactions could contribute to a more favourable binding conformation within the active site. **Compound 27** exhibited fewer interactions overall within the binding site. It formed a hydrogen bond with GLU-802 and engaged in a π-stacking interaction with PHE-349. Overall, the candidate compounds formed a higher number and diversity of interactions, particularly through hydrogen bonds and hydrophobic contacts, suggesting enhanced binding strength compared to **Compound 27**.

### Binding free energy calculation

**Figure 10.**
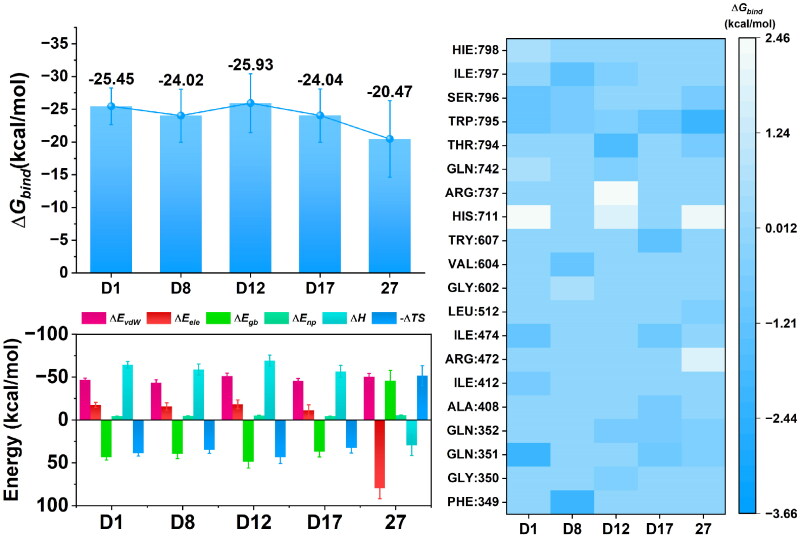
(a) Binding free energy of candidate compounds with RdRp. (b) Energy contribution of residues near the binding site for each complex. (c) Total individual energy terms for each complex.

The binding free energy, individual energy contributions, and residue-specific interactions of the candidate compounds and the reference **Compound 27** were analysed to evaluate their binding affinity towards the RdRp protein (Figure 10 and Tables S4 and S5). These analyses provided insights into the thermodynamic stability and key residue interactions for each complex. The binding free energy values (Figure 10(a)) indicated that all the candidate compounds exhibited stronger binding affinities than that of **Compound 27**. Among the candidates, **D12** demonstrated the most favourable binding free energy at −25.93 kcal/mol, followed by **D1** (−25.45 kcal/mol) and **D8** (−24.02 kcal/mol). In comparison, the reference **Compound 27** showed a higher (less favourable) binding free energy of −20.47 kcal/mol, suggesting weaker binding stability within the binding pocket. The energy contribution analysis (Figure 10(b)) identified several key residues contributing to the binding stability of the complexes. Notably, residues GLY351, TRP795, and SER796 provided substantial stabilising contributions across all candidates. In particular, **D8** benefitted from strong interactions with PHE349 enhancing their binding stability. The heatmap of residue contributions highlighted more favourable interactions for the candidates than that of **Compound 27**, which showed weaker contributions from several critical residues.

The breakdown of individual energy components (Figure 10(c)) revealed that electrostatic interactions (*ΔE_ele_*) and van der Waals forces (*ΔE_vdW_*) were the primary contributors to binding for all the candidates. Notably, **D12** exhibited strong van der Waals interactions, while **D1** benefitted from favourable polar interactions (*ΔE_ele_*). The entropy contribution (TΔS) was slightly more favourable for **Compound 27**, indicating marginally better flexibility, though it did not compensate for its weaker binding interactions overall. The binding free energy and residue-specific interaction analysis demonstrated that the candidate compounds, particularly **D1**, and **D12**, exhibited superior binding stability compared to the reference **Compound 27**. The stronger interactions with key residues, combined with favourable van der Waals and electrostatic energy contributions, highlight the enhanced binding potential of the candidates. In contrast, the weaker binding free energy and suboptimal residue interactions of **Compound 27** further emphasise the advantages of the candidate compounds.

### DFT analysis

#### Structural properties analysis

**Figure 11.**
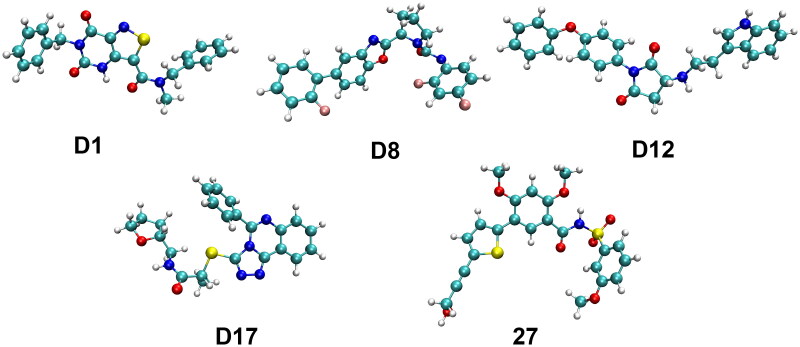
Structural information of candidate compounds and **Compound 27** optimised at the B3LYP/6–311++g(d,p) level.

The structures and geometric properties of the candidate compounds and the reference **Compound 27** were optimised at the **B3LYP/6–311++G(d,p)** level, and their dimensions, volume, and surface areas were calculated to understand their spatial characteristics (Figure 11 and Table 2). These parameters provided insights into the steric factors and molecular surface properties that may influence binding interactions and biological activity. The lengths of the candidate compounds ranged from 17.488 Å (**D8**) to 23.607 Å (**D12**), with **Compound 27** exhibiting a shorter length of 16.908 Å. **D12** was the longest among the candidates, suggesting that it may span a larger binding interface. In terms of width, **D8** had the largest value (17.488 Å), while **Compound 27** measured 13.990 Å. The heights of the candidates varied slightly, with **D12** showing the smallest height (6.789 Å) and **D8** the largest (8.781 Å). For volume, **D12** exhibited the highest value (529.802 Å³), indicating a larger molecular structure, while **D8** had the smallest volume (433.394 Å³). The **Compound 27** displayed a comparable volume of 555.940 Å³. In terms of molecular surface area, **D8** exhibited the largest surface area (491.383 Å^2^), which could enhance its potential for interacting with the target protein. **Compound 27** had a surface area of 501.699 Å^2^, suggesting a slightly larger interface compared to most of the candidate compounds.

**Table 2.** Structural information of compounds **R2**, **R29**, **R37**, **R39**, and **27** calculated at the B3LYP/6–311++g(d,p) level.

Compound	Length (Å)	Width (Å)	Height (Å)	Volume (Å^3^)	Surface area (Å^2^)
**D1**	18.031	8.567	8.567	473.208	411.670
**D8**	17.488	17.488	8.781	433.394	491.383
**D12**	23.607	9.422	6.789	529.802	473.316
**D17**	17.998	11.138	8.247	524.667	457.498
**27**	16.908	13.990	8.035	555.940	501.699

#### MEP analysis

**Figure 12.**
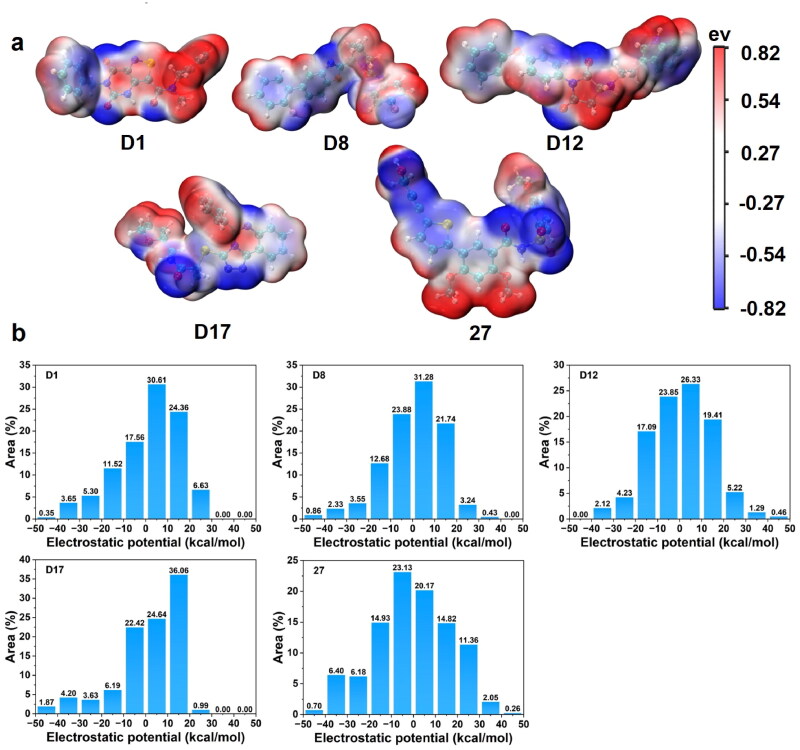
(a) MEP maps of candidate compounds and **Compound 27**. (b) Quantitative distribution of electrostatic potential of candidate compounds and **Compound 27**.

The molecular electrostatic potential (MEP) maps and quantitative distributions of the candidate compounds and the reference **Compound 27** were analysed to assess their electrostatic properties, as shown in Figure 12. The MEP maps provided insights into the distribution of positive and negative charges on the molecular surfaces, which influence intermolecular interactions such as hydrogen bonding, ionic interactions, and binding affinity. The MEP maps (Figure 12(a)) revealed similar charge distributions across the candidate compounds, with red regions indicating electron-rich (negatively charged) areas and blue regions indicating electron-poor (positively charged) areas. Compounds **D1** and **D17** exhibited a larger distribution of negative electrostatic potential, which could enhance their ability to engage in electrostatic interactions with the protein’s positively charged residues. In contrast, **Compound 27** showed a more even distribution of positive and negative regions, reflecting a balanced electrostatic surface.

The quantitative distribution of the electrostatic potential (Figure 12(b)) further highlighted these differences. **D17** exhibited the highest proportion of surface area with positive electrostatic potential, with 36.06% of its area in the range of 0 to −10 kcal/mol, suggesting strong potential for electrostatic interactions. **D1**, **D8**, and **D12** also showed a higher percentage of positive electrostatic potential compared to **Compound 27**, which displayed 20.17% of its surface area in the 0 to −10 kcal/mol range. The candidates generally had more prominent regions with negative potentials, indicating a stronger capacity for forming favourable interactions with the protein’s binding site. The electrostatic analysis indicated that the candidate compounds exhibited larger regions of positive potential compared to **Compound 27**, suggesting that these candidates may have stronger electrostatic interactions within the binding pocket. The **Compound 27**, with a more balanced charge distribution, may have reduced binding efficiency compared to the candidates.

#### Chemical properties analysis

**Figure 13.**
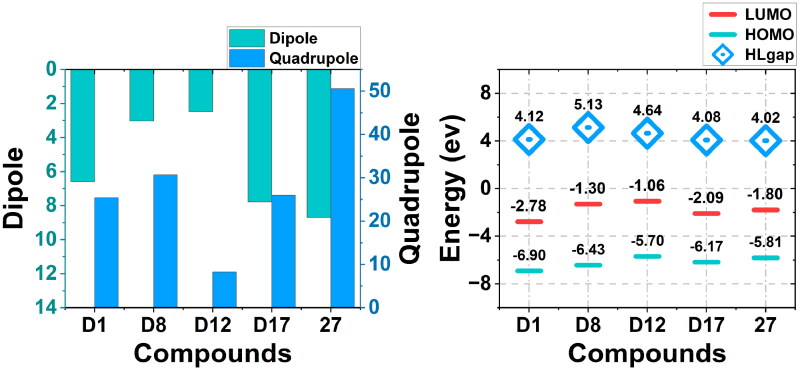
(a) Dipole and quadrupole of candidate compounds and **Compound 27**. (b) LUMO, HOMO, and gap energy (blue square proportional to the gap magnitude) for the ground-state of candidate compounds and **Compound 27**.

The calculation of dipole, quadrupole moments and HOMO-LUMO gap energies was significant because it offered critical insights into a molecule’s chemical stability, reactivity, and interaction potential. In Figure 13(a), the analysis of dipole and quadrupole moments indicated that all candidate compounds exhibited lower values than reference **Compound 27**. This observation suggested that **Compound 27** had a higher polarity and a more significant quadrupole moment compared to the candidate compounds. The reduced dipole and quadrupole moments in the candidates may imply relatively lower polarity and reduced anisotropic interaction potential. These properties could influence the interaction patterns of these compounds, possibly affecting their binding affinity or selectivity in polar or directional interaction environments. For electronic properties, as illustrated in Figure 13(b), the HOMO-LUMO gap energy (indicated by the blue diamonds) was highest in **D8** (5.13 eV), implying a relatively lower reactivity compared to the other compounds. The HOMO and LUMO energies of the candidates spanned from −6.90 eV to −5.70 eV and −2.78 eV to −1.06 eV, respectively. The HOMO-LUMO gap energies of the candidate compounds were consistently higher than that of **Compound 27**. A larger HOMO-LUMO gap generally indicated better chemical stability, as it implied that a greater amount of energy was required to transition the compound from its ground state. This made the candidate compounds less reactive, and thus potentially more stable than the reference compound.

## Conclusions

In this study, a multi-step computational and experimental workflow was implemented to identify novel DENV-NS5 RdRp inhibitors. 27 candidates obtained by virtual screening of 1.6 million compounds from ChemDiv and TargetMol databases through a systematic evaluation using docking score, Uni-Score Rescore, and MM/GBSA binding free energy. To further refine the selection, 50 ns molecular MD simulations were performed, and MM/PBSA analysis revealed that 23 candidates exhibited superior binding free energy compared to the reference **Compound 27**. ADMET evaluations confirmed that the candidates have favourable pharmacokinetic profiles with improved intestinal absorption and permeability, while maintaining acceptable toxicity and clearance levels. Biological activity against DENV-NGC assessments revealed that **D1** and **D8** demonstrated the most potent antiviral activity with IC_50_ values of 13.06 ± 1.17 μM and 14.79 ± 7.76 μM, respectively, outperforming **Compound 27** (IC_50_ = 19.67 ± 1.12 μM). **D12** and **D17** exhibited comparable activity to **BCX4430**. The selectivity index (SI) analysis showed that **D8** had the highest SI (6.71), followed by **D1** (2.87), indicating high selectivity for the virus over host cells. Molecular dynamics simulations were employed to assess the structural stability and dynamic behaviour of the candidates (**D1**, **D8**, **D12** and **D17**) compared to **Compound 27**. The RMSD and Rg analyses revealed that the candidate compounds formed more stable and compact complexes than **Compound 27**. SASA and RMSF analyses indicated that the candidates exhibited solvent exposure and flexibility comparable to **Compound 27**, ensuring stable interactions within the binding pocket. PCA analysis demonstrated that **D1** and **D12** had constrained conformational spaces, indicating rigid and stable binding, while **D8** and **D17** exhibited dynamic behaviour similar to **Compound 27**, reflecting a balance between stability and flexibility. FEL analysis further confirmed the superior stability of **D1** and **D8**, which formed the deepest energy wells, while **D12** and **D17** exhibited energy landscapes similar to **Compound 27**. DCCM analysis revealed stronger coordinated motions for **D1**, **D12**, and **D17**, indicating enhanced stabilisation of residue motions within the complexes. The candidate compounds also exhibited diverse interaction profiles within the active site, forming multiple hydrogen bonds, π-π interactions, and unique halogen bonds with key residues. This range of interactions suggested stronger and more versatile binding compared to the limited interactions observed for **Compound 27**.

Binding free energy calculations revealed that all candidate compounds exhibited stronger binding affinities than **Compound 27**, with **D12** showing the most favourable free energy (−25.93 kcal/mol), followed by **D1** (−25.45 kcal/mol) and **D8** (−24.02 kcal/mol). Key residues such as TRP795, GLY351, and SER796 contributed significantly to binding stability across the candidates. Electrostatic and van der Waals interactions were identified as the primary contributors to binding, with **D12** and **D1** exhibiting particularly strong van der Waals and electrostatic contributions, respectively. DFT analysis provided insights into the structural, electronic, and chemical properties of the candidates. The optimised geometries revealed that **D12** had the largest molecular volume and surface area, potentially spanning a larger binding interface. MEP analysis indicated that **D1** and **D17** exhibited larger regions of negative electrostatic potential, enhancing their potential for forming strong interactions with positively charged residues. The HOMO-LUMO gap analysis demonstrated that the candidates had larger gap energies than **Compound 27**, indicating greater chemical stability and reduced reactivity. Dipole and quadrupole moment calculations confirmed that the candidates had lower polarity and anisotropic interaction potential than **Compound 27**, which may enhance their binding efficiency. The combined computational and experimental analyses presented in this study suggested that the four candidates have great promise for further optimisation and experimental validation as effective antiviral agents, especially as DENV-NS5 RdRp inhibitor. While these computational analyses suggested that the four candidates hold promise as DENV-NS5 RdRp inhibitors, further exploration of their molecular signalling mechanisms was essential^27^. Understanding the detailed molecular interactions between these compounds and host signalling pathways could provide valuable insights into their antiviral mechanisms. For example, evaluating how these compounds influence key molecular pathways involved in immune responses and viral replication, such as the JAK-STAT pathway, may further enhance our understanding of their potential. Future studies could incorporate these mechanistic insights to refine drug design and evaluate their effectiveness in preclinical models of dengue infection. The combined computational and experimental analyses presented in this study suggested that the four candidates have great promise for further optimisation and experimental validation as effective antiviral agents.

## Supplementary Material

Supplementary_Material_ Clean.docx

## Data Availability

Data available on request from the authors.
