# Supplementary material for: Identification of novel inhibitors of dengue viral NS5 RNA-dependent RNA polymerase through molecular docking, biological activity evaluation and molecular dynamics simulations
Source: J Enzyme Inhib Med Chem. 2025 Feb 12;40(1):2463006. doi: 10.1080/14756366.2025.2463006 (PMC11823381; doi:10.1080/14756366.2025.2463006)
Supplement: Supplementary_Material_ Clean.docx [file IENZ_A_2463006_SM9486.docx]

***Supplementary Material***

**Identification of novel inhibitors of dengue viral NS5 RNA-dependent RNA polymerase through molecular docking,** **biological activity evaluation and molecular dynamics simulations**

Keli Zong ^a, b^, Chaochun Wei ^a^, Wei Li ^b^, Cong Wang ^a^, Jiajun Ruan ^b^, Xiaojing Liu ^b^, Susu Zhang ^b^, Hong Yan ^a,^ *, Ruiyuan Cao ^b^ *, and Xingzhou Li ^b,^ *

^a^College of Chemistry and Life Science, Beijing University of Technology, Beijing 100124, P. R. China

^b^Beijing Institute of Pharmacology and Toxicology, 27 Taiping Road, Beijing 100850, China

* Correspondence e-mail: [hongyan@bjut.edu.cn](mailto:hongyan@bjut.edu.cn,), [caoruiyuan@bmi.ac.cn](mailto:caoruiyuan@bmi.ac.cn), and lixz@bmi.ac.cn

**contents**

[Materials and methods 1](#_Toc183606696)

[PCA and DCCM 1](#_Toc183606697)

[Principal component analysis 1](#_Toc183606698)

[Free energy landscape analysis 2](#_Toc183606699)

[Dynamic Cross-Correlation Matrix analysis 2](#_Toc183606700)

[Binding free energy calculation 2](#_Toc183606701)

[Table S1 3](#_Toc183606702)

[Fig. S1 6](#_Toc183606703)

[Fig. S2 7](#_Toc183606704)

[Table S2 8](#_Toc183606705)

[Table S3 9](#_Toc183606706)

[Table S4 11](#_Toc183606707)

[Table S5 11](#_Toc183606708)

# Materials and methods

## PCA and DCCM

## Principal component analysis

Principal Component Analysis (PCA) was performed to investigate the key motions within the DENV-NS5 RdRp-ligand complexes during MD simulations. This method simplifies the multidimensional atomic positional data by identifying collective motions that dominate the protein's dynamic behavior. Using the Cα atoms, a covariance matrix of atomic displacements was generated, where each entry represents the relationship between the movements of two atoms over the simulation. After diagonalizing this matrix, eigenvectors and eigenvalues were obtained, corresponding to the principal components (PCs) and their contribution to the total motion, respectively. The primary PCs, representing the largest variations, were examined to identify significant conformational shifts. By projecting trajectories onto these PCs, key motions within the complexes were visualized. PCA was carried out using GROMACS 2020.7, and the results were visualized with Origin software.

## Free energy landscape analysis

Free Energy Landscape (FEL) analysis was utilized to evaluate the conformational states of DENV RdRp-ligand complexes during MD simulations. FEL offers a thermodynamic perspective by mapping free energy against specific reaction coordinates. In this study, RMSD and Rg were selected as coordinates to represent conformational space. The FEL was constructed to identify stable conformational states, indicated by low-energy basins, and less favorable states, represented by high-energy regions. FELs were generated using the GROMACS 2020.7 software, and visualizations were created with Origin to present the energy landscape comprehensively.

## Dynamic Cross-Correlation Matrix analysis

The Dynamic Cross-Correlation Matrix (DCCM) technique was employed as an effective computational approach to investigate the correlated movements between pairs of atoms in molecular systems, especially for proteins interacting with ligands. The DCCM was generated by computing the cross-correlation coefficients of the displacement vectors for each atomic pair. The DCCM elements, denoted as C_ij_, were determined using the equation:

$$C_{ij}=\frac{\left\langle\delta r_{i}\left( t \right).\delta r_{j}\left( t \right) \right\rangle}{\sqrt{\left\langle\delta r_{i}\left( t \right)^{2} \right\rangle\left\langle\delta r_{j}\left( t \right)^{2} \right\rangle}}$$

For each pair of atoms $i$ and $j$, the displacement vectors $\delta r_{i}\left( t \right)$ and $\delta r_{j}\left( t \right)$ were calculated throughout the simulation, representing the deviations of these atoms from their respective mean positions at any time $t$. These vectors indicated the fluctuations around their average positions during the simulation.

## Binding free energy calculation

The MM-PBSA method was applied to estimate the binding free energy ($\Delta G_{bind}$) of the ligand-protein complexes using the gmx_MMPBSA tool, which integrates functionalities from GROMACS 2020.7 and AmberTools22, streamlining input generation for end-state free energy calculations[1,2]. To ensure the accurate prediction of $\Delta G_{bind}$ and to capture stable conformations between ligands and the RdRp protein, 2500 frames were extracted from the final 5 ns of the 50 ns MD simulations, and 5000 frames were sampled from the last 50 ns of the 200 ns simulations. The $\Delta G_{bind}$ values for the complexes were computed using the following formula:

$$\Delta G_{bind}=\Delta H-T\Delta S$$

$$\Delta H=\Delta G_{sol}+\Delta G_{gas}$$

$$\Delta G_{gas}=\Delta E_{bonded}+\Delta E_{nonbonded}=\left( \Delta E_{bond}+\Delta E_{angle}+\Delta E_{dihedral} \right)+\left( \Delta E_{vdW}+\Delta E_{ele} \right)$$

$$\Delta G_{sol}=\Delta G_{GB}+\Delta G_{SA}=\Delta G_{popar}+\Delta G_{non-polar}$$

$$\Delta G_{non-polar}=\gamma\cdot SASA+\beta$$

Table S1 Score, Uni-score Rescore, and MM/GBSA energy of candidate compounds and **Compound 27**.

| Compound | Structure | Score  (kcal/mol) | Uni-score  Rescore  (kcal/mol) | MM/GBSA  (kcal/mol) |
| --- | --- | --- | --- | --- |
| **D1** |  | -9.215 | -8.805 | -4.739 |
| **D2** |  | -7.988 | -7.168 | -2.087 |
| **D3** |  | -7.015 | -7.172 | -12.668 |
| **D4** |  | -8.970 | -7.730 | *-11.633* |
| **D5** |  | -8.795 | -8.867 | *-2.267* |
| **D6** |  | -7.612 | -7.102 | *-4.836* |
| **D7** |  | -8.546 | -8.359 | *-5.745* |
| **D8** |  | -9.162 | -9.633 | *4.298* |
| **D9** |  | -9.817 | -8.836 | *-2.779* |
| **D10** |  | -7.459 | -7.836 | -4.285 |
| **D11** |  | -9.441 | -8.023 | -2.022 |
| **D12** |  | -10.196 | -9.758 | -6.697 |
| **D13** |  | -8.418 | -7.930 | -8.629 |
| **D14** |  | -9.414 | -8.664 | -2.337 |
| **D15** |  | -7.022 | -7.500 | -5.507 |
| **D16** |  | -9.736 | -7.742 | -3.984 |
| **D17** |  | -6.889 | -7.883 | -2.848 |
| **D18** |  | -8.296 | -7.602 | -2.692 |
| **D19** |  | -7.333 | -8.703 | -2.553 |
| **D20** |  | -6.874 | -7.699 | -7.562 |
| **D21** |  | -10.311 | -8.781 | -2.513 |
| **D22** |  | -7.859 | -8.078 | -11.659 |
| **D23** |  | -7.458 | -7.828 | -2.452 |
| **D24** |  | -7.988 | -8.445 | -2.802 |
| **D25** |  | -8.550 | -7.266 | -9.722 |
| **D26** |  | -7.966 | -7.355 | -2.888 |
| **D27** |  | -7.025 | -7.461 | -12.175 |
| **27** |  | -8.512 | -7.062 | -2.578 |


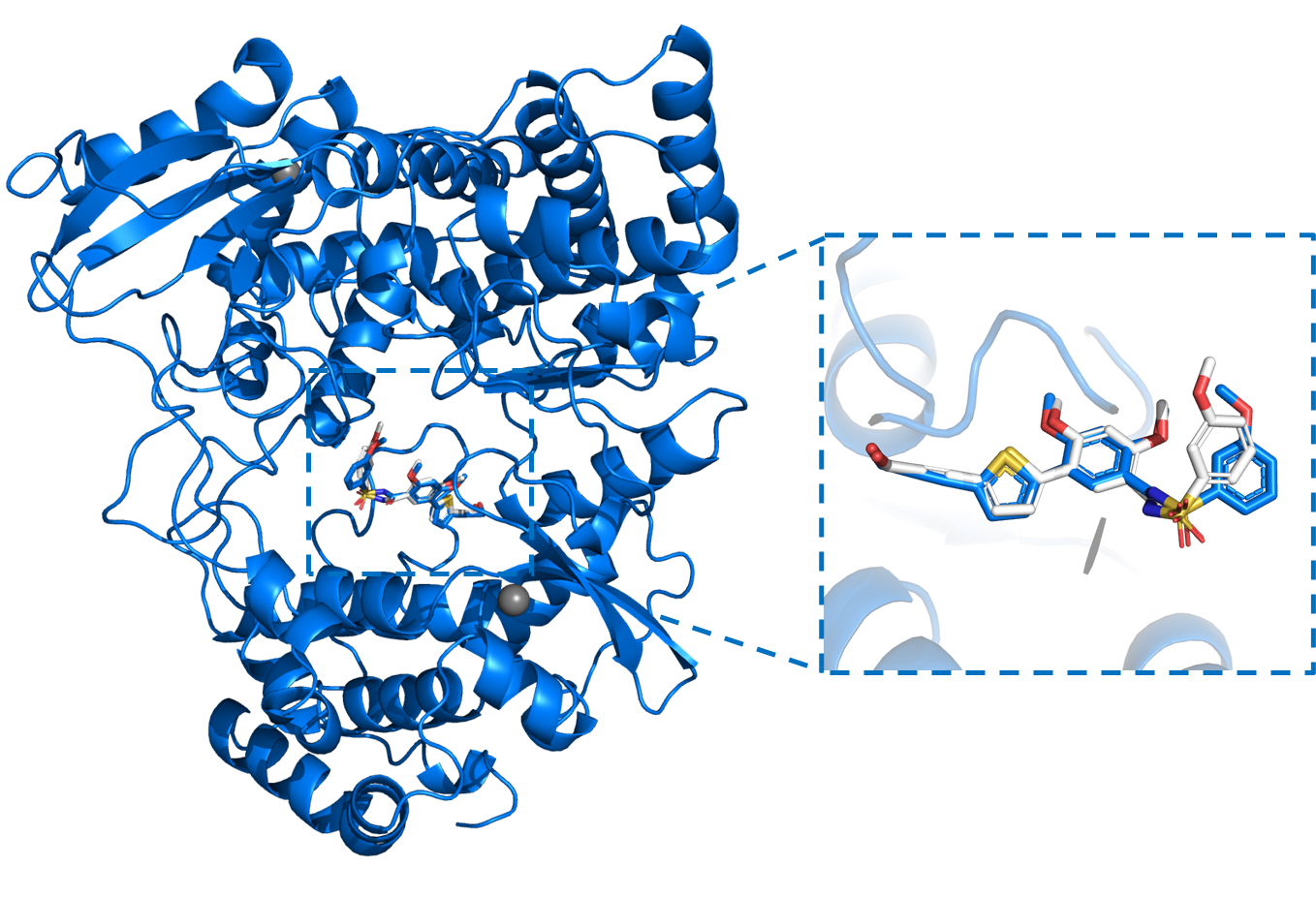


Fig. S1 Alignment of redocked (white) and crystallographic (blue) ligand in RdRp active site validating docking precision.


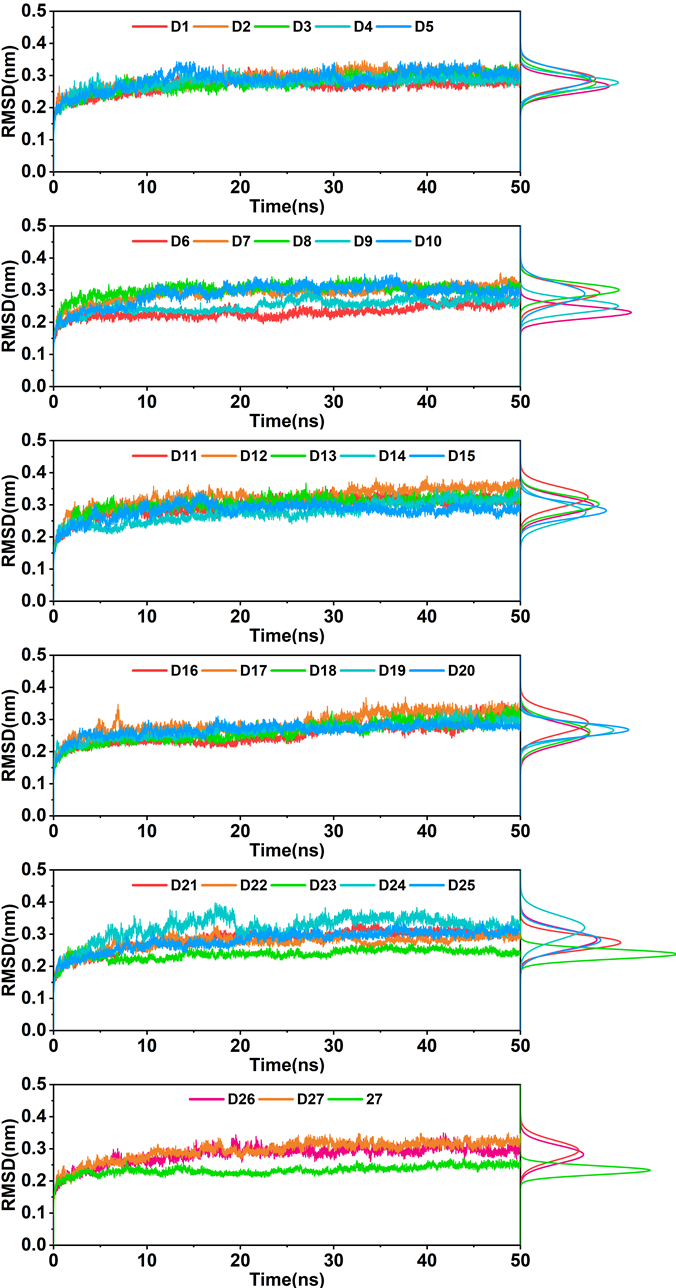


Fig. S2 RMSD of candidate compounds and **Compound 27** complexes over 50 ns

Table S2 Binding free energy and each energy terms of the candidate compounds and **Compound 27** with DENV-NS5 RdRp over 50 ns

| Compound | **Δ*E_vdW_*** | **Δ*E_ele_*** | **Δ*G_gb_*** | **Δ*G_np_*** | **Δ*G_gb_*** | **-TΔ*S*** | **Δ*G_bing_*** |
| --- | --- | --- | --- | --- | --- | --- | --- |
| **D1** | -47.10±2.78 | -16.33±4.03 | 44.60±3.06 | -4.72±0.10 | -63.44±4.55 | 39.88±3.03 | -23.55±3.89 |
| **D2** | -28.88±1.90 | 223.34±11.03 | -177.11±10.38 | 3.46±0.13 | 194.45±11.02 | -180.57±10.38 | 13.89±5.29 |
| **D3** | -43.11±2.14 | 223.28±10.72 | -184.39±9.64 | -4.73±0.12 | 180.17±10.68 | -189.12±9.63 | -8.95±4.17 |
| **D4** | -54.50±3.25 | 164.45±9.20 | -134.32±8.11 | -5.62±0.14 | 109.95±9.89 | -139.94±8.10 | -29.99±4.38 |
| **D5** | -48.03±2.38 | -12.77±5.06 | 48.09±6.23 | -4.90±0.09 | -60.80±5.31 | 43.19±6.22 | -17.61±6.91 |
| **D6** | -53.32±2.71 | -32.84±7.65 | 66.56±5.87 | -4.85±0.12 | -86.16±8.12 | 61.70±5.83 | -24.46±4.55 |
| **D7** | -52.92±2.62 | -39.18±6.80 | 69.21±8.68 | -4.92±0.10 | -92.09±7.58 | 64.30±8.66 | -27.80±4.49 |
| **D8** | -48.35±2.21 | -2.08±4.11 | 38.54±3.86 | -4.78±0.11 | -50.43±4.57 | 33.76±3.85 | -16.66±3.42 |
| **D9** | -45.13±2.34 | -20.12±3.67 | 43.80±3.86 | -4.26±0.09 | -65.26±4.24 | 39.54±3.82 | -25.71±3.04 |
| **D10** | -58.11±2.81 | -35.93±4.72 | 64.91±5.05 | -5.77±0.14 | -94.04±5.18 | 59.14±4.99 | -34.90±3.95 |
| **D11** | -43.53±2.25 | -12.96±3.73 | 49.63±4.57 | -4.52±0.08 | -56.49±4.48 | 45.11±4.57 | -11.37±3.55 |
| **D12** | -56.41±2.68 | -26.33±4.21 | 53.62±4.19 | -5.38±0.11 | -82.74±4.67 | 48.24±4.16 | -34.50±3.95 |
| **D13** | -42.33±4.93 | 209.78±19.27 | -177.42±20.71 | -4.58±0.23 | 167.45±23.01 | -182.00±20.56 | -14.55±4.37 |
| **D14** | -50.55±3.48 | -34.95±7.39 | 55.09±4.02 | -4.88±0.10 | -85.50±8.74 | 50.21±4.05 | -35.29±6.06 |
| **D15** | -30.16±1.80 | -0.78±5.38 | 18.30±5.31 | -2.98±0.10 | -30.95±5.82 | 15.32±5.28 | -15.62±2.33 |
| **D16** | -57.84±2.57 | -22.80±4.70 | 53.18±4.22 | -4.89±0.10 | -80.64±4.94 | 48.29±4.23 | -32.35±3.44 |
| **D17** | -45.85±3.32 | -10.49±5.84 | 43.61±4.90 | -4.58±0.19 | -56.33±7.67 | 39.03±4.82 | -17.30±5.92 |

| **D18** | -36.12±2.13 | 228.13±10.15 | -196.96±9.86 | -3.73±0.21 | 192.00±10.28 | -200.69±9.83 | -8.68±3.46 |
| --- | --- | --- | --- | --- | --- | --- | --- |
| **D19** | -46.44±3.17 | -9.97±3.67 | 41.34±3.82 | -5.40±0.19 | -56.41±4.22 | 35.94±3.76 | -20.47±3.70 |
| **D20** | -39.10±2.11 | -0.22±2.69 | 30.19±3.27 | -4.04±0.16 | -39.31±2.86 | 26.14±3.29 | -13.17±3.09 |
| **D21** | -39.04±2.20 | -19.98±4.40 | 39.21±3.90 | -3.93±0.15 | -59.02±4.51 | 35.28±3.85 | -23.74±2.80 |
| **D22** | -27.22±2.39 | 228.45±11.51 | -200.51±11.95 | -3.17±0.25 | 201.23±12.43 | -203.68±11.86 | -2.45±3.72 |
| **D23** | -43.23±3.05 | 2.78±6.42 | 30.00±4.17 | -4.38±0.15 | -40.45±5.95 | 25.61±4.19 | -14.83±4.30 |
| **D24** | -44.56±2.78 | -5.14±4.21 | 40.30±5.95 | -4.95±0.16 | -49.71±5.30 | 35.35±5.87 | -14.35±3.90 |
| **D25** | -40.90±1.88 | 266.60±7.44 | -221.65±6.88 | -4.21±0.10 | 225.71±7.44 | -225.86±6.88 | -0.15±4.09 |
| **D26** | -45.90±2.65 | -12.40±7.89 | 33.19±7.50 | -4.58±0.11 | -58.30±8.30 | 28.61±7.46 | -29.69±3.81 |
| **D27** | -33.63±2.08 | 224.73±8.56 | -185.21±7.71 | -3.31±0.08 | 191.10±8.32 | -188.52±7.71 | 2.58±3.86 |
| **27** | -56.77±3.00 | 202.08±8.29 | -147.29±9.41 | -5.60±0.12 | 145.31±8.62 | -152.89±9.39 | -7.58±6.86 |

Table S3 ADMET properties of the candidate compounds **BCX4430**, **NITD008**, and **Compound 27**.

| **Compound** | **Water solubility**  **(log mol/L)** | **Caco2 permeability**  **(log Papp in 10^-6^ cm/s)** | **Intestinal absorption (%)** | **VDss (log L/kg)** | **CYP2C19 inhibitior** | **CYP2C9 inhibitior** | **CYP2D6 inhibitior** | **Total Clearance**  **(Log ml/min/kg)** | **hERG I inhibitor** | **hERG II inhibitor** | **Oral Rat Chronic Toxicity**  **(log mg/kg_bw/day)** | **Hepatotoxicity** | **Skin Sensitization** |
| --- | --- | --- | --- | --- | --- | --- | --- | --- | --- | --- | --- | --- | --- |
| **D1** | -3.60 | 0.84 | 95.85 | -0.43 | Yes | Yes | No | -0.02 | No | Yes | 1.22 | No | No |
| **D3** | -4.04 | 0.53 | 90.81 | -1.03 | No | No | No | 0.22 | No | No | 1.02 | Yes | No |
| **D4** | -4.61 | 1.06 | 91.83 | 0.40 | No | No | No | 0.53 | No | Yes | 1.57 | Yes | No |
| **D5** | -4.68 | 0.99 | 91.92 | -0.47 | Yes | No | No | -0.025 | No | Yes | 0.54 | No | No |
| **D6** | -5.21 | 0.47 | 83.76 | -0.49 | No | Yes | No | 0.041 | No | No | 0.20 | Yes | No |
| **D7** | -4.09 | 0.031 | 77.04 | -0.70 | No | No | No | 0.50 | No | No | 1.80 | Yes | No |
| **D8** | -4.2 | 1.29 | 89.78 | -0.46 | Yes | Yes | No | -0.05 | No | Yes | 1.33 | Yes | No |
| **D9** | -4.48 | -0.021 | 73.05 | -1.10 | No | No | No | -0.41 | No | Yes | 1.76 | Yes | No |
| **D10** | -4.32 | 0.80 | 93.49 | 0.10 | No | No | No | 0.58 | No | Yes | -0.068 | No | No |
| **D11** | -3.81 | 1.06 | 93.71 | 0.17 | Yes | Yes | No | 0.31 | No | Yes | 1.19 | Yes | No |
| **D12** | -4.51 | 0.68 | 92.89 | 0.47 | Yes | Yes | Yes | 1.14 | No | Yes | 1.29 | Yes | No |
| **D13** | -3.93 | 0.79 | 90.41 | 0.56 | No | No | Yes | 0.35 | No | Yes | 0.92 | Yes | No |
| **D14** | -3.31 | 0.19 | 79.33 | -0.27 | Yes | Yes | No | -0.39 | No | Yes | 2.31 | Yes | No |
| **D15** | -2.18 | 0.90 | 73.86 | -0.01 | No | No | No | 0.31 | No | No | 1.89 | Yes | No |
| **D16** | -3.72 | 0.55 | 89.57 | -0.33 | Yes | Yes | No | -0.077 | No | Yes | 0.86 | Yes | No |
| **D17** | -3.72 | 1.14 | 96.73 | 0.12 | Yes | Yes | No | 0.41 | No | Yes | 0.21 | Yes | No |
| **D18** | -2.27 | -0.034 | 50.53 | -1.27 | No | No | No | 1.28 | No | No | 0.91 | Yes | No |
| **D19** | -4.16 | 1.119 | 89.16 | 0.27 | Yes | Yes | No | -0.22 | No | Yes | 1.66 | Yes | No |
| **D20** | -4.49 | 1.231 | 94.00 | 0.15 | Yes | Yes | No | -0.012 | No | No | 1.18 | Yes | No |

Table S5 Energy contribution of residues near the binding site for candidate compounds and **Compound 27** complex

|  | PHE:349 | GLY:350 | GLN:351 | GLN:352 | ALA:408 | ILE:412 | ARG:472 | ILE:474 | LEU:512 | GLY:602 | VAL:604 | TRY:607 | HIS:711 | ARG:737 | GLN:742 | THR:794 | TRP:795 | SER:796 |
| --- | --- | --- | --- | --- | --- | --- | --- | --- | --- | --- | --- | --- | --- | --- | --- | --- | --- | --- |
| **D1** | 0 | 0 | -2.11 | 0 | 0 | -0.64 | 0 | -1.11 | 0 | 0 | 0 | 0 | 2.45 | 0 | 0.58 | 0 | -1.17 | -1.06 |
| **D8** | -2.22 | 0 | 0 | 0 | 0 | 0 | 0 | 0 | 0 | 0.58 | -1.06 | 0 | 0 | 0 | 0 | 0 | -0.78 | -0.79 |
| **D12** | 0 | -0.6 | 0 | -0.73 | 0 | 0 | 0 | 0 | 0 | 0 | 0 | 0 | 1.84 | 2.3 | -0.55 | -1.67 | -0.57 | 0 |
| **D17** | 0 | 0 | -0.89 | -0.75 | -0.67 | 0 | 0 | -0.91 | 0 | 0 | 0 | -1.38 | 0 | 0 | 0 | 0 | -1.06 | 0 |
| **27** | 0 | 0 | -0.58 | -0.57 | 0 | 0 | 1.84 | 0 | -0.52 | 0 | 0 | 0 | 2.06 | 0 | 0 | -0.7 | -2.05 | -0.67 |

Table S4 Binding free energy and each energy terms of the candidate compounds and **Compound 27** with DENV-NS5 RdRp over 200ns

| Compound | **Δ*E_vdW_*** | **Δ*E_ele_*** | **Δ*G_gb_*** | **Δ*G_np_*** | **Δ*G_gb_*** | **-TΔ*S*** | **Δ*G_bing_*** |
| --- | --- | --- | --- | --- | --- | --- | --- |
| **D1** | -46.71±2.19 | -17.61±2.96 | 43.39±3.48 | -4.53±0.11 | -64.32±3.89 | 38.87±3.45 | -25.45±2.81 |
| **D8** | -43.4±3.49 | -15.6±4.39 | 39.71±5.48 | -4.73±0.28 | -59.01±6.28 | 34.98±4.05 | -24.02±4.05 |
| **D12** | -51.14±3.43 | -18.14±5.13 | 48.69±7.58 | -5.34±0.17 | -69.28±6.45 | 43.35±7.5 | -25.93±4.49 |
| **D17** | -45.63±2.67 | -11.08±6.47 | 37.11±6.06 | -4.44±0.18 | -56.71±6.94 | 32.67±6.04 | -24.04±4.07 |
| **27** | -50.34±3.9 | 79.92±12.07 | -45.92±11.79 | -5.61±0.14 | 29.58±12.11 | -51.53±11.77 | -20.47±5.84 |

| **D21** | -5.39 | 0.54 | 94.38 | -0.27 | Yes | Yes | No | -0.30 | No | Yes | 0.30 | Yes | No |
| --- | --- | --- | --- | --- | --- | --- | --- | --- | --- | --- | --- | --- | --- |
| **D23** | -4.54 | 0.92 | 87.44 | 0.11 | Yes | No | No | 0.30 | No | No | 1.88 | Yes | No |
| **D24** | -4.82 | 0.97 | 92.7 | 0.46 | Yes | Yes | No | 0.55 | No | Yes | 1.14 | No | No |
| **D26** | -4.79 | 0.99 | 91.45 | 0.35 | Yes | Yes | No | -0.028 | No | Yes | 1.14 | Yes | No |
| **BCX4430** | -2.48 | 0.51 | 53.42 | 0.72 | No | No | No | 1.06 | No | No | 2.74 | Yes | No |
| **27** | -4.79 | -0.03 | 81.39 | -0.54 | Yes | Yes | No | 0.50 | No | Yes | 1.15 | Yes | No |

**References**

[1] B.R. Miller, T.D. Mcgee, J.M. Swails, N. Homeyer, H. Gohlke, A.E. Roitberg, MMPBSA.py: an efficient program for end-state free energy calculations, J. Chem. Theory Comput. 8 (2012) 3314. 10.1021/ct300418h.

[2] M.S. Valdés-Tresanco, M.E. Valdés-Tresanco, P.A. Valiente, E. Moreno, Gmx_MMPBSA: a new tool to perform end-state free energy calculations with GROMACS, J. Chem. Theory Comput. 17 (2021) 6281. 10.1021/acs.jctc.1c00645.
